# The leap to ordinal: Detailed functional prognosis after traumatic brain injury with a flexible modelling approach

**DOI:** 10.1371/journal.pone.0270973

**Published:** 2022-07-05

**Authors:** Shubhayu Bhattacharyay, Ioan Milosevic, Lindsay Wilson, David K. Menon, Robert D. Stevens, Ewout W. Steyerberg, David W. Nelson, Ari Ercole, the CENTER-TBI investigators participants

**Affiliations:** 1 Division of Anaesthesia, University of Cambridge, Cambridge, United Kingdom; 2 Department of Clinical Neurosciences, University of Cambridge, Cambridge, United Kingdom; 3 Laboratory of Computational Intensive Care Medicine, Johns Hopkins University, Baltimore, MD, United States of America; 4 Division of Psychology, University of Stirling, Stirling, United Kingdom; 5 Department of Anesthesiology and Critical Care Medicine, Johns Hopkins University, Baltimore, MD, United States of America; 6 Department of Biomedical Data Sciences, Leiden University Medical Center, Leiden, The Netherlands; 7 Department of Physiology and Pharmacology, Section for Perioperative Medicine and Intensive Care, Karolinska Institutet, Stockholm, Sweden; 8 Cambridge Centre for Artificial Intelligence in Medicine, Cambridge, United Kingdom; Columbia University, UNITED STATES

## Abstract

When a patient is admitted to the intensive care unit (ICU) after a traumatic brain injury (TBI), an early prognosis is essential for baseline risk adjustment and shared decision making. TBI outcomes are commonly categorised by the Glasgow Outcome Scale–Extended (GOSE) into eight, ordered levels of functional recovery at 6 months after injury. Existing ICU prognostic models predict binary outcomes at a certain threshold of GOSE (e.g., prediction of survival [GOSE > 1]). We aimed to develop ordinal prediction models that concurrently predict probabilities of each GOSE score. From a prospective cohort (*n* = 1,550, 65 centres) in the ICU stratum of the Collaborative European NeuroTrauma Effectiveness Research in TBI (CENTER-TBI) patient dataset, we extracted all clinical information within 24 hours of ICU admission (1,151 predictors) and 6-month GOSE scores. We analysed the effect of two design elements on ordinal model performance: (1) the baseline predictor set, ranging from a concise set of ten validated predictors to a token-embedded representation of all possible predictors, and (2) the modelling strategy, from ordinal logistic regression to multinomial deep learning. With repeated *k*-fold cross-validation, we found that expanding the baseline predictor set significantly improved ordinal prediction performance while increasing analytical complexity did not. Half of these gains could be achieved with the addition of eight high-impact predictors to the concise set. At best, ordinal models achieved 0.76 (95% CI: 0.74–0.77) ordinal discrimination ability (ordinal *c*-index) and 57% (95% CI: 54%– 60%) explanation of ordinal variation in 6-month GOSE (Somers’ *D*_*xy*_). Model performance and the effect of expanding the predictor set decreased at higher GOSE thresholds, indicating the difficulty of predicting better functional outcomes shortly after ICU admission. Our results motivate the search for informative predictors that improve confidence in prognosis of higher GOSE and the development of ordinal dynamic prediction models.

## Introduction

Globally, traumatic brain injury (TBI) is a major cause of death, disability, and economic burden [1]. The treatment of critically ill TBI patients is largely guided by an initial prognosis made within a day of admission to the intensive care unit (ICU) [2]. Early outcome prediction models set a baseline against which clinicians consider the effect of therapeutic strategies and compare patient trajectories. Therefore, well-calibrated and reliable prognostic models are an essential component of intensive care.

Outcome after TBI is most often evaluated on the ordered, eight-point Glasgow Outcome Scale–Extended (GOSE) [3–6], which stratifies patients by their highest level of functional recovery according to participation in daily activities. Existing baseline prediction models used in the ICU dichotomise the GOSE into binary endpoints for TBI outcome. For example, the Acute Physiologic Assessment and Chronic Health Evaluation (APACHE) II [7] model predicts in-hospital survival (GOSE > 1) while the International Mission for Prognosis and Analysis of Clinical Trials in TBI (IMPACT) [8] models focus on predicting functional independence (GOSE > 4, or ‘favourable outcome’) and survival at 6 months post-injury.

**Fig 1:**
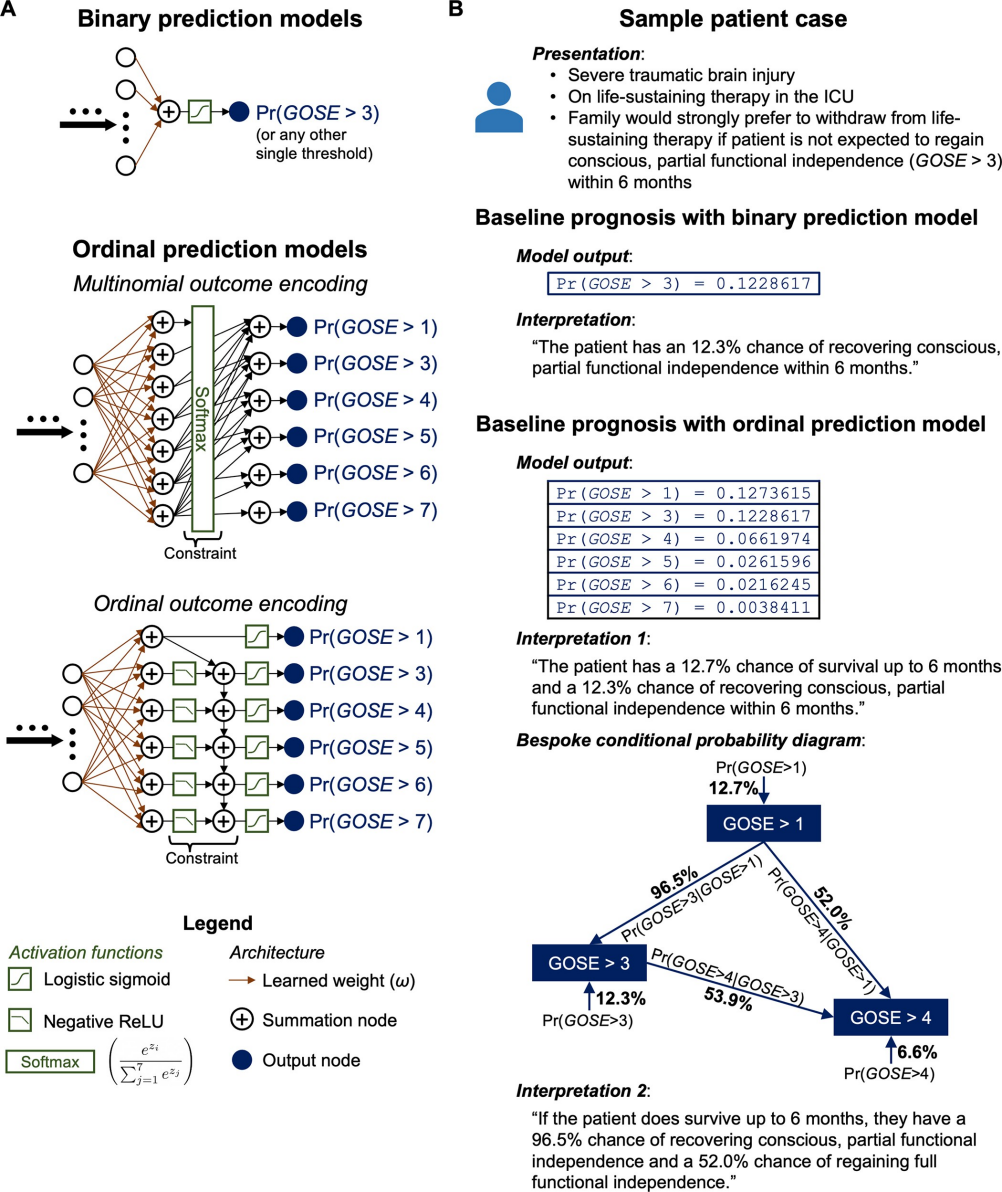
Comparison of ordinal outcome prediction to binary outcome prediction in terms of model architecture and clinical application. GOSE = Glasgow Outcome Scale–Extended at 6 months post-injury. ReLU = rectified linear unit. Pr(●) = Probability operator, i.e., “probability of ●.” Pr(●|○) = Conditional probability operator, i.e., “probability of ●, given ○.” (**A**) Output layer architectures of binary and ordinal GOSE prediction models. Ordinal prediction models must not only have a more complicated output structure (in terms of learned weights and outcome encoding choices) but also constrain probabilities across the possible levels of functional outcome (indicated by ‘Constraint’ in the ordinal model representations). The constraint for multinomial outcome encoding is performed with a softmax activation function while the constraint for ordinal outcome encoding is performed with subtractions of output values (implemented with a negative ReLU transformation) from lower thresholds. In the provided legend formula for the softmax activation function, *z*_*i*_ represents the outputted value of the *i*^*th*^ node of the multinomial outcome encoding layer (i.e., the node representing the *i*^*th*^ possible score of GOSE) preceding the softmax transformation. (**B**) A sample patient case to demonstrate the difference in prognostic information between ordinal and binary GOSE prediction models. Binary models predict outcomes at one GOSE threshold while ordinal models predict outcomes at every GOSE threshold concurrently and provide conditional predictions of higher GOSE threshold outcomes given lower GOSE threshold outcomes. Bespoke conditional probability diagrams can be constructed between any number of GOSE thresholds, as desired by model users, so long as lower thresholds (e.g., GOSE > 1) precede higher thresholds (e.g., GOSE > 3) in directionality. Conditional probabilities are calculated by dividing the model probability at the higher threshold by the model probability at the lower threshold (e.g., Pr(GOSE>3|GOSE>1)=Pr(GOSE>3)/Pr(GOSE>1)).

Dichotomised GOSE prediction employs a fixed threshold of favourability among the eight levels of recovery for all patients. However, there is no empirical justification for an ideal treatment-effect threshold of GOSE [9]. Moreover, dichotomisation removes each patient or caregiver’s ability to define a different level of recovery as ‘favourable’ during prognosis. By concealing the nuanced differences in outcome defined by the GOSE, dichotomisation also limits the prognostic information made available during a shared treatment decision making process. For example, when clinicians, patients, or next of kin must together decide whether to withdraw life-sustaining measures (WLSM) after severe TBI, knowing the probability of different levels of functional recovery in addition to the baseline probability of survival would enable better quality-of-life consideration and confidence in the decision (**Fig 1B**) [10]. These problems of dichotomisation cannot be addressed simply by independently training a combination of binary prediction models at several GOSE thresholds. If model predictions are not constrained across the thresholds (i.e., ensuring probabilities do not increase with higher thresholds) during training, then combining multiple threshold outputs may result in nonsensical values. For example, the purported probability of survival (GOSE > 1) might be lower than that of recovering functional independence (GOSE > 4).

A practical solution would be to train ordinal outcome prediction models, which concurrently return probabilities at each GOSE threshold by learning the interdependent relationships between the predictor set and the possible levels of functional recovery (**Fig 1A**). Ordinal GOSE prediction models would allow users to interpret the probability of different levels of functional recovery. Additionally, they can provide insight into the conditional probability of obtaining greater levels of recovery given lower levels (see **Fig 1B** for a practical clinical application of this information). However, moving from binary to ordinal outcome prediction poses three key challenges. First, there is no guarantee that widely accepted TBI outcome predictor sets, validated either by binary or ordinal regression analysis, will be able to capture the nuanced differences between levels of functional recovery well enough for reliable prediction. Second, ordinal prediction models typically need to be more complicated than binary models to encode the possibility of more outcomes and the constrained relationship between them [11]. For GOSE prediction, ordinal models can either encode the outcomes as: (1) multinomial, in which nodes exist for each GOSE score and collectively undergo a softmax transformation (to constrain the sum of values to one) and probabilities are calculated by accumulating values up to each threshold, or (2) ordinal, in which nodes exist for each threshold between consecutive GOSE scores, constrained such that output values must not increase with higher thresholds, and probabilities for each threshold are calculated with a sigmoid transformation (**Fig 1A**). Third, assessment of prediction performance is not as intuitive with an ordinal outcome as with a binary outcome. Widely used dichotomous prediction performance metrics such as the *c*-index (i.e., the area under the receiver operating characteristic curve [AUC]) do not trivially extend to the ordinal case [12], so assessment of ordinal prediction models requires the consideration of multifactorial metrics and visualisations that may complicate interpretations of model performance [13].

As part of the Collaborative European NeuroTrauma Effectiveness Research in TBI (CENTER-TBI) project, we aim to address the challenges of ordinal outcome prediction. Our analyses cover a range of modelling strategies and predictors available within the first 24 hours of admission to the ICU.

## Materials and methods

### Study population and dataset

The study population was extracted from the ICU stratum of the core CENTER-TBI dataset (v3.0) using Opal database software [14]. The project objectives and experimental design of CENTER-TBI have been described in detail by Maas *et al*. [15] and Steyerberg *et al*. [16] Study patients were prospectively recruited at one of 65 participating ICUs across Europe with the following eligibility criteria: admission to the hospital within 24 hours of injury, indication for CT scanning, and informed consent according to local and national requirements.

Per project protocol, each patient’s follow-up schedule included a GOSE assessment at 6 months post-injury, or, more precisely, within a window of 5–8 months post-injury. GOSE assessments were conducted using structured interviews [6] and patient/carer questionnaires [17] by the clinical research team of CENTER-TBI. The eight, ordinal scores of GOSE, representing the highest levels of functional recovery, are decoded in the heading of **Table 1**. Since patient/carer questionnaires do not distinguish vegetative patients (GOSE = 2) into a separate category, GOSE scores 2 and 3 (lower severe disability) were combined to one category (GOSE ∈ {2,3}) in our dataset. Of the 2,138 ICU patients in the CENTER-TBI dataset available for analysis, we excluded patients in the following order: (1) age less than 16 years at ICU admission (*n* = 82), (2) follow-up GOSE was unavailable (*n* = 283), and (3) ICU stay was less than 24 hours (*n* = 223). Our resulting sample size was *n* = 1,550. For 1,351 patients (87.2%), either the patient died during ICU stay (*n* = 205) or results from a GOSE evaluation at 5–8 months post-injury were available in the dataset (*n* = 1,146). For the remaining 199 patients (12.8%), GOSE scores were imputed using a Markov multi-state model based on the observed GOSE scores recorded at different timepoints between 2 weeks to one-year post-injury [18]. A flow diagram for study inclusion and follow-up is provided in **S1 Fig**, and summary characteristics of the study population are detailed in **Table 1**.

**Table 1:** Summary characteristics of the study population at ICU admission stratified by ordinal 6-month outcomes.

Summary characteristics		Overall	Glasgow Outcome Scale–Extended (GOSE) at 6 months post-injury							*p-*value^‡^
			(1) Death	(2 or 3) Vegetative or lower severe disability	(4) Upper severe disability	(5) Lower moderate disability	(6) Upper moderate disability	(7) Lower good recovery	(8) Upper good recovery	
*n* *		1550	318 (20.5%)	262 (16.9%)	120 (7.7%)	227 (14.6%)	200 (12.9%)	206 (13.3%)	217 (14.0%)	
Age [years]		51 (31–66)	66 (50–76)	55 (36–68)	48 (29–61)	44 (31–56)	41 (27–53)	48 (31–65)	41 (24–61)	<0.0001
Sex										0.59
	Female	409 (26.4%)	78 (24.5%)	71 (27.1%)	43 (35.8%)	64 (28.2%)	49 (24.5%)	59 (28.6%)	45 (20.7%)	
Race (*n*^†^ = 1427)										0.13
	White	1386 (97.1%)	281 (97.2%)	239 (96.8%)	106 (95.5%)	195 (96.5%)	183 (97.3%)	184 (98.4%)	198 (97.5%)	
	Black	21 (1.5%)	2 (0.7%)	4 (1.6%)	3 (2.7%)	5 (2.5%)	3 (1.6%)	2 (1.1%)	2 (1.0%)	
	Asian	20 (1.4%)	6 (2.1%)	4 (1.6%)	2 (1.8%)	2 (1.0%)	2 (1.1%)	1 (0.5%)	3 (1.5%)	
Baseline GCS (*n*^†^ = 1465)		8 (4–14)	5 (3–10)	6 (3–10)	8 (4–13)	8 (5–13)	9 (6–14)	13 (7–15)	13 (8–15)	<0.0001
	Mild [13–15]	390 (26.6%)	30 (10.3%)	38 (15.3%)	26 (23.4%)	42 (19.5%)	66 (34.9%)	91 (45.3%)	97 (46.4%)	
	Moderate [9–12]	331 (22.6%)	65 (22.3%)	41 (16.5%)	28 (25.2%)	65 (30.2%)	36 (19.0%)	40 (19.9%)	56 (26.8%)	
	Severe [3–8]	744 (50.8%)	196 (67.4%)	170 (68.3%)	57 (51.4%)	108 (50.2%)	87 (46.0%)	70 (34.8%)	56 (26.8%)	

Data are median (IQR) for continuous characteristics and *n* (% of column group) for categorical characteristics, unless otherwise indicated. Units or numerical definitions of characteristics are provided in square brackets. Baseline GCS = Glasgow Coma Scale at ICU admission, from 3 to 15. Conventionally, TBI severity is categorically defined by baseline GCS scores as indicated in square brackets.

*Percentages for sample size (*n*) represent proportion of study sample size in each GOSE group.

^†^Limited sample size of non-missing values for characteristic.

^‡^*p*-values are determined from proportional odds logistic regression (POLR) coefficient analysis trained on all summary characteristics concurrently [19]. For categorical variables with *k* > 2 categories (e.g., Race), *p*-values were calculated with a likelihood ratio test (with *k*-1 degrees of freedom) on POLR.

### Repeated *k*-fold cross-validation

We implemented the ‘scikit-learn’ module (v0.23.2) [20] in Python (v3.7.6) to create 100 stratified partitions of our study population for repeated *k*-fold cross-validation (20 repeats, 5 folds). Within each of the partitions, approximately 80% of the population would constitute the training set (*n* ≈ 1,240 patients) and 20% of the population would constitute the corresponding testing set (*n* ≈ 310 patients). For parametric (i.e., deep learning) models, we implemented a stratified shuffle split on each of the 100 training sets to set 15% (*n* ≈ 46 patients) aside for validation and hyperparameter optimisation.

### Selection and preparation of concise predictor set

In selecting a concise predictor set, our primary aim was to find a small group of well-validated, widely measured clinical variables that are commonly used for TBI outcome prognosis in existing ICU practice. We selected the ten predictors from the extended IMPACT binary prediction model [8] for moderate-to-severe TBI–defined by a baseline Glasgow Coma Scale (GCS) [21, 22] score between 3 and 12, inclusive–to represent our concise set. While 26.6% of our study population falls out of this GCS range (**Table 1**), we find that the IMPACT predictor set is the most rigorously validated [23–27] baseline set available for the overall critically ill TBI population. The ten predictors, characterised in **Table 2**, are all measured within 24 hours of ICU admission and include demographic characteristics, clinical severity scores, CT characteristics, and laboratory measurements. The predictors as well as empirical justification for their inclusion in the IMPACT model have been described in detail [28]. In this manuscript, each of the models trained on the IMPACT predictor set is denoted as a concise-predictor-based model (CPM).

**Table 2:** Concise baseline predictors of the study population stratified by ordinal 6-month outcomes.

Concise predictors		Overall (*n* = 1550)	Glasgow Outcome Scale–Extended (GOSE) at 6 months post-injury							*p-*value^‡^
			1 (*n* = 318)	2 or 3 (*n* = 262)	4 (*n* = 120)	5 (*n* = 227)	6 (*n* = 200)	7 (*n* = 206)	8 (*n* = 217)	
Age [years]		51 (31–66)	66 (50–76)	55 (36–68)	48 (29–61)	44 (31–56)	41 (27–53)	48 (31–65)	41 (24–61)	<0.0001
GCSm (*n*^†^ = 1509)		5 (1–6)	2 (1–5)	3 (1–5)	5 (1–6)	5 (1–6)	5 (2–6)	5 (3–6)	6 (5–6)	<0.0001
	(1) No response	484 (32.1%)	152 (50.0%)	104 (40.6%)	35 (29.9%)	63 (28.5%)	46 (23.6%)	47 (23.0%)	37 (17.5%)	
	(2) Abnormal extension	54 (3.6%)	17 (5.6%)	20 (7.8%)	4 (3.4%)	6 (2.7%)	3 (1.5%)	2 (1.0%)	2 (0.9%)	
	(3) Abnormal flexion	63 (4.2%)	14 (4.6%)	12 (4.7%)	8 (6.8%)	11 (5.0%)	8 (4.1%)	4 (2.0%)	6 (2.8%)	
	(4) Withdrawal from stimulus	114 (7.6%)	27 (8.9%)	23 (9.0%)	8 (6.8%)	20 (9.0%)	21 (10.8%)	8 (3.9%)	7 (3.3%)	
	(5) Movement localised to stimulus	305 (20.2%)	52 (17.1%)	47 (18.4%)	24 (20.5%)	50 (22.6%)	46 (23.6%)	44 (21.6%)	42 (19.8%)	
	(6) Obeys commands	489 (32.4%)	42 (13.8%)	50 (19.5%)	38 (32.5%)	71 (32.1%)	71 (36.4%)	99 (48.5%)	118 (55.7%)	
Unreactive pupils (*n*^†^ = 1465)										<0.0001
	One	111 (7.6%)	31 (10.5%)	31 (12.3%)	7 (6.3%)	20 (9.3%)	5 (2.6%)	8 (4.1%)	9 (4.4%)	
	Two	168 (11.5%)	84 (28.5%)	33 (13.0%)	8 (7.2%)	14 (6.5%)	8 (4.2%)	16 (8.2%)	5 (2.4%)	
Hypoxia		207 (13.4%)	60 (18.9%)	33 (12.6%)	14 (11.7%)	35 (15.4%)	33 (16.5%)	16 (7.8%)	16 (7.4%)	0.37
Hypotension		210 (13.5%)	56 (17.6%)	51 (19.5%)	21 (17.5%)	32 (14.1%)	22 (11.0%)	15 (7.3%)	13 (6.0%)	0.0038
Marshall CT (*n*^†^ = 1255)		VI (II–VI)	III (II–VI)	II (II–VI)	II (II–VI)	II (II–II)	II (II–III)	II (II–II)	VI (II–VI)	0.043
	No visible pathology (I)	118 (9.4%)	8 (3.3%)	11 (5.3%)	5 (5.2%)	17 (8.7%)	25 (15.2%)	24 (13.6%)	28 (16.5%)	
	Diffuse injury II	592 (47.2%)	56 (22.8%)	84 (40.6%)	54 (56.2%)	92 (47.2%)	100 (60.6%)	103 (58.5%)	103 (60.6%)	
	Diffuse injury III	108 (8.6%)	42 (17.1%)	17 (8.2%)	10 (10.4%)	14 (7.2%)	9 (5.5%)	6 (3.4%)	10 (5.9%)	
	Diffuse injury IV	16 (1.3%)	7 (2.8%)	1 (0.5%)	1 (1.0%)	4 (2.1%)	1 (0.6%)	1 (0.6%)	1 (0.6%)	
	Mass lesion (V & VI)	421 (33.5%)	133 (54.0%)	94 (45.4%)	26 (27.1%)	68 (34.9%)	30 (18.2%)	42 (23.9%)	28 (16.5%)	
tSAH (*n*^†^ = 1254)		957 (76.3%)	221 (90.2%)	176 (84.2%)	73 (76.0%)	150 (76.9%)	106 (63.9%)	125 (71.4%)	106 (63.1%)	0.16
EDH (*n*^†^ = 1257)		244 (19.4%)	31 (12.7%)	32 (15.3%)	21 (21.9%)	46 (23.6%)	32 (19.3%)	42 (23.9%)	40 (23.5%)	0.016
Glucose [mmol/L] (*n*^†^ = 1062)		7.7 (6.6–9.4)	8.8 (7.3–11)	8.0 (6.5–9.8)	7.6 (6.5–9.3)	7.8 (6.6–9.6)	7.7 (6.5–8.7)	7.3 (6.3–8.5)	7.1 (6.3–8.1)	0.013
Hb [g/dL] (*n*^†^ = 1140)		13 (12–14)	13 (11–14)	13 (11–14)	14 (12–14)	13 (12–14)	14 (12–15)	13 (12–15)	14 (13–15)	0.038

Data are median (IQR) for continuous characteristics and *n* (% of column group) for categorical characteristics. Units of characteristics are provided in square brackets. GCSm = motor component score of the Glasgow Coma Scale. Marshall CT = Marshall computerised tomography classification. tSAH = traumatic subarachnoid haemorrhage. EDH = extradural haematoma. Hb = haemoglobin.

^†^Limited sample size of non-missing values for characteristic.

^‡^*p*-values are determined from proportional odds logistic regression (POLR) analysis trained on all concise predictors concurrently [19] and are combined across 100 missing value imputations via *z*-transformation [29]. For categorical variables with *k* > 2 categories (e.g., GCSm), *p*-values were calculated with a likelihood ratio test (with *k*-1 degrees of freedom) on POLR.

Seven of the concise predictors had missing values for some of the patients in our study population (**S2 Fig**). In each repeated cross-validation partition, we trained an independent, stochastic predictive mean matching imputation function on the training set and imputed all missing values across both sets using the ‘mice’ package (v3.9.0) [30] in R (v4.0.0) [31]. The result was a multiply imputed (*m* = 100) dataset with a unique imputation per partition, allowing us to simultaneously account for the variability due to resampling and the variability due to missing value imputation during repeated cross-validation.

Prior to the training of CPMs, each of the multi-categorical variables (i.e., GCSm, Marshall CT, and unreactive pupils in **Table 2**) were one-hot encoded and each of the continuous variables (i.e., age, glucose, and haemoglobin) were standardised based on the mean and standard deviation of each of the training sets with the ‘scikit-learn’ module in Python.

### Selection of concise-predictor-based models (CPMs)

We tested four CPM types, each denoted by a subscript: (1) multinomial logistic regression (CPM_MNLR_), (2) proportional odds (i.e., ordinal) logistic regression (CPM_POLR_), (3) class-weighted feedforward neural network with a multinomial (i.e., softmax) output layer (CPM_DeepMN_), and (4) class-weighted feedforward neural network with an ordinal (i.e., constrained sigmoid at each threshold) output layer (CPM_DeepOR_). These models were selected because, in the setting of ordinal GOSE prediction, we wished to compare the performance of: (1) nonparametric logistic regression models (CPM_MNLR_ and CPM_POLR_) to nonlinear, parametric deep learning networks (CPM_DeepMN_ and CPM_DeepOR_), and (2) multinomial outcome encoding (CPM_MNLR_ and CPM_DeepMN_) to ordinal outcome encoding (CPM_POLR_ and CPM_DeepOR_). Each of these model types returns a predicted probability for each of the GOSE thresholds at 6 months post-injury from the concise set of predictors (**Fig 1A**). A detailed explanation of CPM architectures, hyperparameters for the parametric CPMs, loss functions, and optimisation algorithms is provided in **S1 Appendix**.

CPM_Best_ denotes the optimal CPM for a given performance metric in the **Results**. CPM_MNLR_ and CPM_POLR_ were implemented with the ‘statsmodels’ module (dev. v0.14.0) [32] in Python, and CPM_DeepMN_ and CPM_DeepOR_ were implemented with the ‘PyTorch’ (v1.10.0) [33] module in Python.

### Design of all-predictor-based models (APMs)

In contrast to the CPMs, we designed and trained prediction models on all baseline (i.e., available to ICU clinicians at 24 hours post-admission) clinical information (excluding high-resolution data such as full brain images or physiological waveforms) in the CENTER-TBI database. Each of these models is designated as an all-predictor-based model (APM).

For our study population, there are 1,151 predictors [34], each being in one of the 14 categories listed in **Table 3**, with variable levels of missingness and frequency per patient. This information also includes 81 predictors denoting treatments or interventions within the first 24 hours of ICU care (e.g., type and dose of medication administered) and 76 predictors denoting the explicit impressions or rationales of ICU physicians (e.g., reason for surgical intervention and expected prognosis with or without surgery).

**Table 3:** Predictor baseline tokens per patient in the CENTER-TBI dataset.

Predictor category	Types of tokens				
	All	Fixed at ICU admission	Continuous variable	Treatments and interventions	Physician impression or rationale
Emergency care and ICU admission	112 (103–121)	112 (103–121)	13 (10–16)	0 (0–0)	7 (7–8)
Brain imaging	94 (72–114)	74 (68–83)	5 (2–8)	0 (0–0)	9 (8–10)
ICU monitoring and management	63 (52–72)	3 (3–3)	10 (5–13)	40 (34–46)	13 (3–15)
Injury characteristics and severity	55 (49–62)	55 (49–62)	2 (2–2)	0 (0–0)	0 (0–0)
End-of-day assessments	50 (45–54)	0 (0–0)	19 (17–21)	0 (0–0)	0 (0–0)
Laboratory measurements	44 (32–55)	14 (0–20)	42 (31–52)	0 (0–0)	1 (1–1)
Medical and behavioural history	38 (32–51)	38 (32–51)	0 (0–1)	0 (0–0)	0 (0–0)
Medications	30 (21–40)	0 (0–0)	0 (0–0)	22 (15–30)	8 (5–11)
Bihourly assessments	17 (0–32)	0 (0–0)	15 (0–27)	1 (0–2)	0 (0–0)
Demographics and socioeconomic status	15 (14–16)	15 (14–16)	2 (1–2)	0 (0–0)	0 (0–0)
Protein biomarkers	5 (5–5)	0 (0–0)	5 (5–5)	0 (0–0)	0 (0–0)
Surgery	2 (1–6)	1 (1–2)	0 (0–0)	0 (0–1)	1 (0–3)
Haemostatic markers*	0 (0–0)	0 (0–0)	0 (0–0)	0 (0–0)	0 (0–0)
Transitions of care*	0 (0–0)	0 (0–0)	0 (0–0)	0 (0–0)	0 (0–0)
**All predictors**	532 (486–580)	315 (288–341)	111 (90–132)	64 (50–75)	37 (29–44)

Data represent median (IQR) number of non-missing, unique tokens per patient. Tokens were extracted from the clinical information available up to 24 hours after ICU admission for each study patient in the Collaborative European NeuroTrauma Effectiveness Research in TBI (CENTER-TBI) project dataset. Each token may be of only one predictor category (leftmost column) and of any number of token types (four rightmost columns). ICU = intensive care unit.

*Due to their relative infrequency in the CENTER-TBI dataset, these baseline predictor categories have a 3^rd^ quartile of zero tokens per patient.

**Fig 2:**
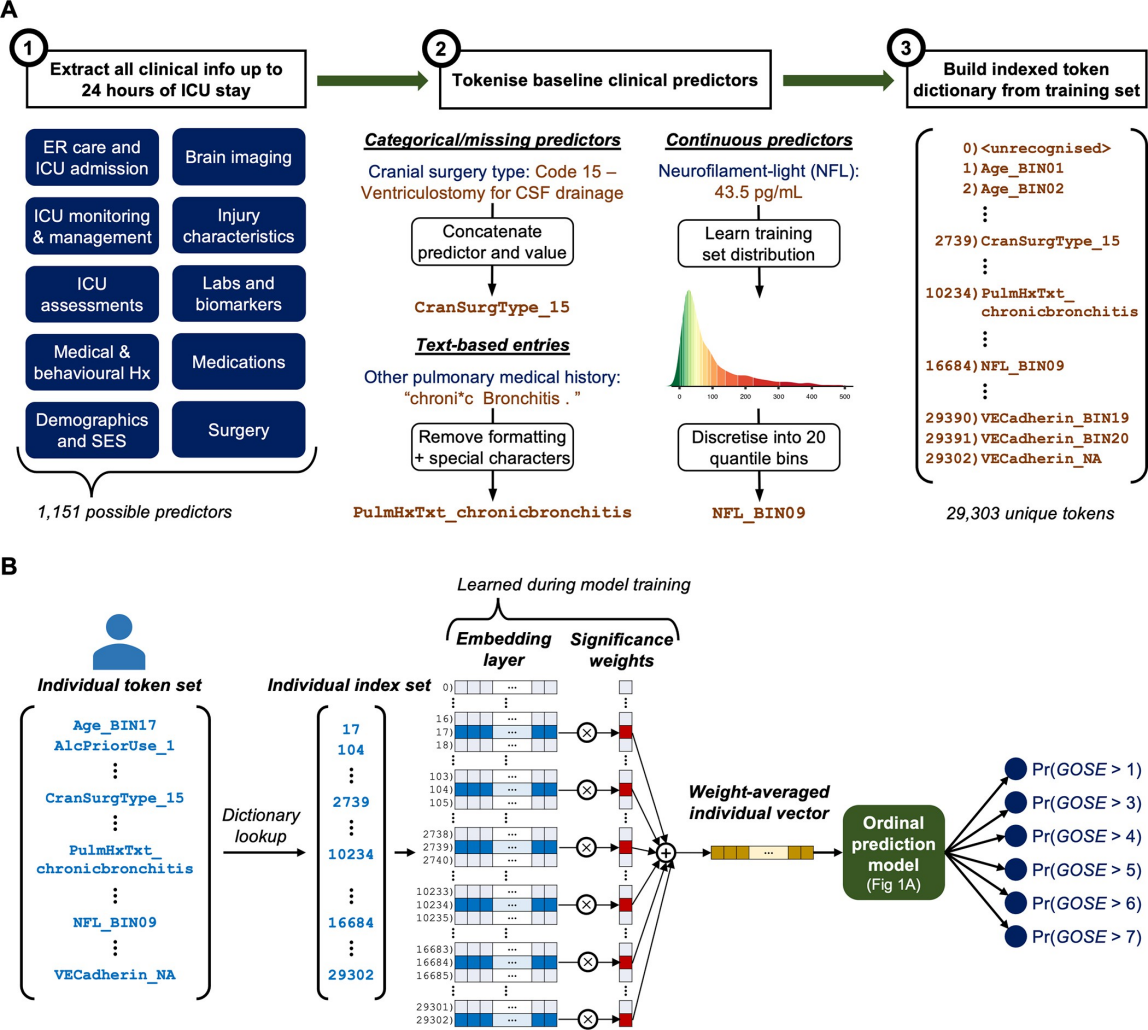
Tokenisation and embedding procedure for the development of ordinal all-predictor-based models (APMs). ICU = intensive care unit. ER = emergency room. Hx = history. SES = socioeconomic status. CSF = cerebrospinal fluid. GOSE = Glasgow Outcome Scale–Extended at 6 months post-injury. (**A**) Process of converting all clinical information, from the first 24 hours of each patient, into an indexed dictionary of tokens during model training. The tokenisation process is illustrated with three example predictors and their associated values in step 2. The first entry in the trained token dictionary (‘0) <unrecognised>‘) of step 3 is a placeholder token for any tokens encountered in the testing set that were not seen in the training set. (**B**) Visual representation of token embedding and significance-weighted averaging pipeline during APM prediction runs. After tokenising an individual patient’s clinical information, the vector of tokens is converted to a vector of the indices corresponding to each token in the trained token dictionary. The corresponding vectors and significance weights of the indices are extracted to weight-average the patient information into a single vector. The embedding layer and significance weights are learned through stochastic gradient descent during model training, and significance weights are constrained to be positive with an exponential function. While not explicitly shown, the weighted vectors are divided by the number of vectors during weight-averaging. The individual, weight-averaged vector then feeds into an ordinal prediction model to return probabilities at each GOSE threshold. The ordinal prediction model could either have multinomial output encoding (APM_MN_) or ordinal outcome encoding (APM_OR_), as represented in **Fig 1A**.

To prepare this information into a suitable format for training APMs, we tokenised and embedded heterogenous patient data [35] in a process visualised in **Fig 2**. Predictor tokens were constructed in one of the following ways: (1) for categorical predictors, a token was constructed by concatenating the predictor name and value, e.g., ‘GCSTotalScore_04,’ (2) for continuous predictors, a token was constructed by learning the distribution of that predictor from the training set and discretising into 20 quantile bins, e.g., ‘SystolicBloodPressure_BIN17,’ (3) for text-based entries, we removed all special characters, spaces, and capitalisation from the text and appended the unformatted text to the predictor name, e.g., ‘InjuryDescription_skullfracture,’ and (4) for missing values, a separate token was created to designate missingness, e.g., ‘PriorMedications_NA’ (**Fig 2A**). The unique tokens from a patient’s first 24 hours of ICU stay made up his or her individual predictor set, and the median number of unique tokens (excluding missing value tokens) per patient per predictor category are provided in **Table 3**. Notably, this process does not require any data cleaning, missing value imputation, outlier removal, or domain-specific knowledge for a large set of variables and imposes no constraints on the number or type of predictors per patients [35]. Additionally, by including missing value tokens, models can discover meaningful patterns of missingness if they exist [36].

Taking inspiration from artificially intelligent (AI) natural language processing [37, 38], all the predictor tokens from the training set (excluding the validation set) are used to construct a token dictionary. APMs learn a lower dimensional vector as well as a positive significance weight for each entry in the dictionary during training. The vectors for each of the tokens of a single patient are significance-weight-averaged into a single vector which is then fed into a class-weighted feedforward neural network (**Fig 2B**). If the neural network has no hidden layers, then the APM is analogous to logistic regression, while if it does have hidden layers, the APM corresponds to deep learning. In this work, we train APMs with one of two kinds of output layers: multinomial, i.e., softmax, (APM_MN_), or ordinal, i.e., constrained sigmoid at each GOSE threshold, (APM_OR_). Both model types output a predicted probability for each of the GOSE thresholds at 6 months post-injury. A detailed explanation of APM architectures, hyperparameters, loss functions, and optimisation algorithms is provided in **S2 Appendix**.

APM_Best_ denotes the optimal APM for a given performance metric in the **Results**. APM_MN_ and APM_OR_ were implemented with the ‘PyTorch’ module in Python.

### Predictor importance in all-predictor-based models (APMs)

The relative importance of predictor tokens in the trained APMs was measured with absolute Shapley additive explanation (SHAP) [39] values, which, in our case, can be interpreted as the magnitude of the relative contribution of a token towards a model output for a single patient. For APM_MN_, this corresponds to the predictor contributions towards each node (after softmax transformation, **Fig 1A**) corresponding to the probability at a GOSE score. For APM_OR_, this corresponds to the predictor contributions towards each node (after sigmoid transformation, **Fig 1A**) corresponding to the probability at a GOSE threshold. Absolute SHAP values were measured for each patient in the testing set of every repeated cross-validation partition, and we averaged these values over the partitions to derive our individualised importance scores per token. These scores were averaged, once again, over the entire patient set to calculate the mean absolute SHAP values of each token. Finally, to derive importance scores for each predictor, we calculated the maximum of the mean absolute SHAP values of the possible tokens from the predictor.

### Selection and preparation of extended concise predictor set

We selected a small set of the most important APM predictors by mean absolute SHAP values to add to the concise predictor set and observe the change in model performance. Since the concise predictor set does not include any information on intervention decisions or physician impressions from the first day, we did not consider these predictor types. Moreover, for every multi-categorical predictor selected, we examined the mean absolute SHAP values of each of the predictor’s possible tokens to determine which of the categories should be explicitly encoded (e.g., including 10 categories for employment status or just one indicator variable for retirement). The extended concise predictor set, including the 10 original concise predictors and the 8 added predictors, in our study population is listed and characterised in **S1 Table**. Each of the models trained on the concise set with these variables added is denoted as an extended concise-predictor-based model (eCPM).

The process of multiple imputation (*m* = 100), one-hot encoding, and standardisation of the extended concise predictor set was identical to that of the concise predictor set, as described earlier.

### Selection of extended concise-predictor-based models (eCPMs)

The four eCPM model types we tested are identical to the four CPM model types, as described earlier and in **S1 Appendix** with, however, the extended concise predictor set: (1) multinomial logistic regression (eCPM_MNLR_), (2) proportional odds (i.e., ordinal) logistic regression (eCPM_POLR_), (3) class-weighted feedforward neural network with a multinomial (i.e., softmax) output layer (eCPM_DeepMN_), and (4) class-weighted feedforward neural network with an ordinal (i.e., constrained sigmoid at each threshold) output layer (eCPM_DeepOR_).

eCPM_Best_ denotes the optimal eCPM for a given performance metric in the **Results**.

### Assessment of model discrimination and calibration

All model metrics, curves, and associated confidence intervals (CI) were calculated from testing set predictions using the repeated Bootstrap Bias Corrected Cross-Validation (BBC-CV) method [40] with 1,000 resamples of unique patients for bootstrapping. The collection of metrics from the bootstrapped testing set resamples for each model then formed our unbiased estimation distribution for statistical inference (i.e., CI).

In this work, we assess model discrimination performance (i.e., how well do the models separate patients with different GOSE scores?) and probability calibration (i.e., how reliable are the predicted probabilities at each threshold?). The metrics and visualisations are explained in detail, with mathematical derivation and intuitive examples, in **S3 Appendix**. In this section, we will only list the metrics, their interpretations, and their range of feasible values. Feasible values range from the value corresponding to no model information or random guessing (i.e., the no information value [NIV]) to the value corresponding to ideal model performance (i.e., the full information value [FIV]).

Our primary metric of model discrimination performance is the ordinal *c*-index (ORC) [13]. ORC has two interpretations: (1) the probability that a model correctly separates two patients with two randomly chosen GOSE scores and (2) the average proportional closeness between a model’s functional outcome ranking of a set of patients (which includes one randomly chosen patient from each possible GOSE score) to their true functional outcome ranking. In addition, we calculate Somers’ *D*_*xy*_ [41, 42], which is interpreted as the proportion of ordinal variation in GOSE that can be explained by the variation in model output. Our final metrics of model discrimination are dichotomous *c*-indices (i.e., AUC) at each threshold of GOSE. Each is interpreted as the probability of a model correctly discriminating a patient with GOSE above the threshold from one with GOSE below. The range of feasible values for each discrimination metric are: NIV_ORC_ = 0.5 to FIV_ORC_ = 1, NIV_Somers’ *Dxy*_ = 0 to FIV_Somers’ *Dxy*_ = 1, and NIV_Dichotomous *c*-index_ = 0.5 to FIV_Dichotomous *c*-index_ = 1. ORC is the only discrimination metric that is independent of the sample prevalence of each GOSE category [13].

To assess the calibration of predicted probabilities at each GOSE threshold, we use the logistic recalibration framework [43] to measure calibration slope [44]. A calibration slope less than one indicates overfitting (i.e., high predicted probabilities are overestimated while low predicted probabilities are underestimated) while a calibration slope greater than one indicates underfitting [45]. We also examine smoothed probability calibration curves [46] to detect miscalibrations that may be overlooked by the logistic recalibration framework [45]. The ideal calibration curve is a diagonal line with slope one and *y*-intercept 0 while one indicative of random guessing would be a horizontal line with a *y*-intercept at the proportion of the study population above the given threshold. We accompany each calibration curve with the integrated calibration index (ICI) [47], which is the mean absolute error between the smoothed and the ideal calibration curves, to aid comparison of curves across model types. FIV_ICI_ = 0, but NIV_ICI_ varies based on the outcome distribution at each threshold (**S3 Appendix**).

All metrics were calculated using the ‘scikit-learn’ and ‘SciPy’ (v1.6.2) [48] modules in Python and figures were plotted using the ‘ggplot2’ package (v3.3.2) [49] in R.

### Computational resources

All computational and statistical components of this work were performed in parallel on the Cambridge Service for Data Driven Discovery (CSD3) high performance computer, operated by the University of Cambridge Research Computing Service (http://www.hpc.cam.ac.uk). The training of each APM was accelerated with graphical processing units and the ‘PyTorch Lightning’ (v1.5.0) [50] module. The training of all parametric models (CPM_DeepMN_, CPM_DeepOR_, APM_MN_, APM_OR_, eCPM_DeepMN_, and eCPM_DeepOR_) was made more efficient by dropping out consistently underperforming parametric configurations, on the validation sets, with the Bootstrap Bias Corrected with Dropping Cross-Validation (BBCD-CV) method [40] with 1,000 resamples of unique patients. The results of hyperparameter optimisation are detailed in **S4 Appendix**.

## Results

### CPM and APM discrimination performance

The discrimination performance metrics for each CPM are listed in **S2 Table**. Deep learning models (CPM_DeepMN_ and CPM_DeepOR_) made no significant improvement (based on 95% CI) over logistic regression models (CPM_MNLR_ and CPM_POLR_). The only significant difference in discrimination among the model types was observed in CPM_DeepOR_, which had a significantly lower ORC and Somers’ *D*_*xy*_ than the other models. The discrimination performance metrics for each APM are listed in **S3 Table**. APM_MN_ had a significantly higher ORC, Somers’ *D*_*xy*_, and dichotomous *c*-indices at lower GOSE thresholds (i.e., GOSE > 1 and GOSE > 3) than did APM_OR_. Moreover, in **S4 Appendix**, we see that the best-performing parametric configurations of APM_MN_ did not contain additional hidden layers between the token embedding and output layers. Our results of performance within predictor sets consistently demonstrate that increasing analytical complexity, in terms of using deep learning (for CPMs) or adding hidden network layers (for APMs), did not improve discrimination of outcomes. In the case of deep learning models, multinomial outcome encoding significantly outperformed ordinal outcome encoding (**Fig 1A**).

The discrimination performance metrics of the best-performing CPMs (CPM_Best_), compared with those of the best-performing APMs (APM_Best_), are listed in **Table 4**. In contrast to the case of analytical complexity, we observe that expanding the predictor set yielded a significant improvement in ORC, Somers’ *D*_*xy*_, and each threshold-level dichotomous *c*-index except for those of the highest GOSE thresholds (i.e., GOSE > 6 and GOSE > 7). On average, models trained on the concise predictor set (CPMs) correctly separated two randomly selected patients from two randomly selected GOSE categories 70% (95% CI: 68%– 71%) of the time, while models trained on all baseline predictors (APMs) in the CENTER-TBI dataset did so 76% (95% CI: 74%– 77%) of the time. These percentages also correspond to the average proportional closeness of predicted rankings to true GOSE rankings of patient sets. CPM_Best_ explained 44% (95% CI: 41%– 48%) of the ordinal variation in GOSE while APM_Best_ explained 57% (95% CI: 54%– 60%) in their respective model outputs. At increasing GOSE thresholds, the dichotomous *c*-indices of CPM_Best_ and APM_Best_, as well as the gap between them, consistently decreased (**Table 4**). This signifies that predicting higher 6-month functional outcomes is more difficult than predicting lower 6-month functional outcomes. Moreover, the gains in discrimination earned from expanding the predictor set mostly come from improved performance at lower GOSE thresholds (i.e., predicting survival, return of consciousness, or recovery of functional independence).

**Table 4:** Best ordinal model discrimination and calibration performance per predictor set.

Metric	Threshold	Model		
		CPM_Best_	APM_Best_	eCPM_Best_
Ordinal *c*-index (ORC)		0.70 (0.68–0.71)	0.76 (0.74–0.77)	0.73 (0.71–0.74)
Somers’ *D*_*xy*_		0.44 (0.41–0.48)	0.57 (0.54–0.60)	0.50 (0.46–0.54)
Threshold-level dichotomous *c*-index*		0.77 (0.75–0.78)	0.82 (0.80–0.83)	0.79 (0.78–0.80)
	GOSE > 1	0.83 (0.81–0.85)	0.90 (0.88–0.92)	0.86 (0.84–0.87)
	GOSE > 3	0.81 (0.79–0.83)	0.86 (0.84–0.88)	0.84 (0.83–0.86)
	GOSE > 4	0.78 (0.76–0.80)	0.83 (0.80–0.85)	0.82 (0.80–0.83)
	GOSE > 5	0.76 (0.74–0.77)	0.80 (0.78–0.83)	0.77 (0.75–0.79)
	GOSE > 6	0.72 (0.70–0.74)	0.76 (0.73–0.79)	0.75 (0.73–0.77)
	GOSE > 7	0.72 (0.69–0.74)	0.75 (0.72–0.79)	0.72 (0.70–0.75)
Threshold-level calibration slope*		0.98 (0.81–1.12)	0.84 (0.76–0.91)	1.00 (0.78–1.14)
	GOSE > 1	0.95 (0.78–1.10)	0.98 (0.86–1.10)	0.98 (0.78–1.14)
	GOSE > 3	0.97 (0.80–1.12)	0.90 (0.80–1.02)	1.05 (0.81–1.20)
	GOSE > 4	1.06 (0.86–1.23)	0.89 (0.79–1.00)	1.10 (0.85–1.27)
	GOSE > 5	1.01 (0.78–1.21)	0.82 (0.72–0.94)	1.01 (0.76–1.22)
	GOSE > 6	0.98 (0.73–1.20)	0.74 (0.62–0.87)	0.97 (0.70–1.20)
	GOSE > 7	0.92 (0.69–1.18)	0.68 (0.54–0.83)	0.89 (0.61–1.18)

Data represent mean (95% confidence interval) for the best-performing model, per predictor set, based on a given metric. For threshold-level metrics, a single best-performing model, per predictor set, was determined by the overall unweighted average across the thresholds. Interpretations for each metric are provided in **Materials and methods**. Mean and confidence interval values were derived using bias-corrected bootstrapping (1,000 resamples) and represent the variation across repeated *k*-fold cross-validation folds (20 repeats of 5 folds) and, for the concise-predictor-based model (CPM) and the extended concise-predictor-based model (eCPM), 100 missing value imputations. CPM_Best_ = CPM with best value for given metric (**S2 Table**). APM_Best_ = all-predictor-based model (APM) with best value for given metric (**S3 Table**). eCPM_Best_ = eCPM with best value for given metric (**S4 Table**). GOSE = Glasgow Outcome Scale–Extended at 6 months post-injury.

*Values in these rows correspond to the unweighted average across all GOSE thresholds.

### CPM and APM calibration performance

The calibration slopes and calibration curves for each CPM are displayed in **S2 Table** and **S3 Fig**, respectively. Both logistic regression CPMs (CPM_MNLR_ and CPM_POLR_) are significantly overfitted at the three highest GOSE thresholds (i.e., GOSE > 5, GOSE > 6, and GOSE > 7). The graphical calibration of CPM_DeepOR_ was significantly worse than that of the other CPMs (**S3 Fig**). The calibration slopes and calibration curves for each APM are displayed in **S3 Table** and **S4 Fig**, respectively. APM_OR_ is poorly calibrated at each threshold of GOSE. APM_MN_ is significantly overfitted at the three highest GOSE thresholds (i.e., GOSE > 5, GOSE > 6, and GOSE > 7).

**Fig 3:**
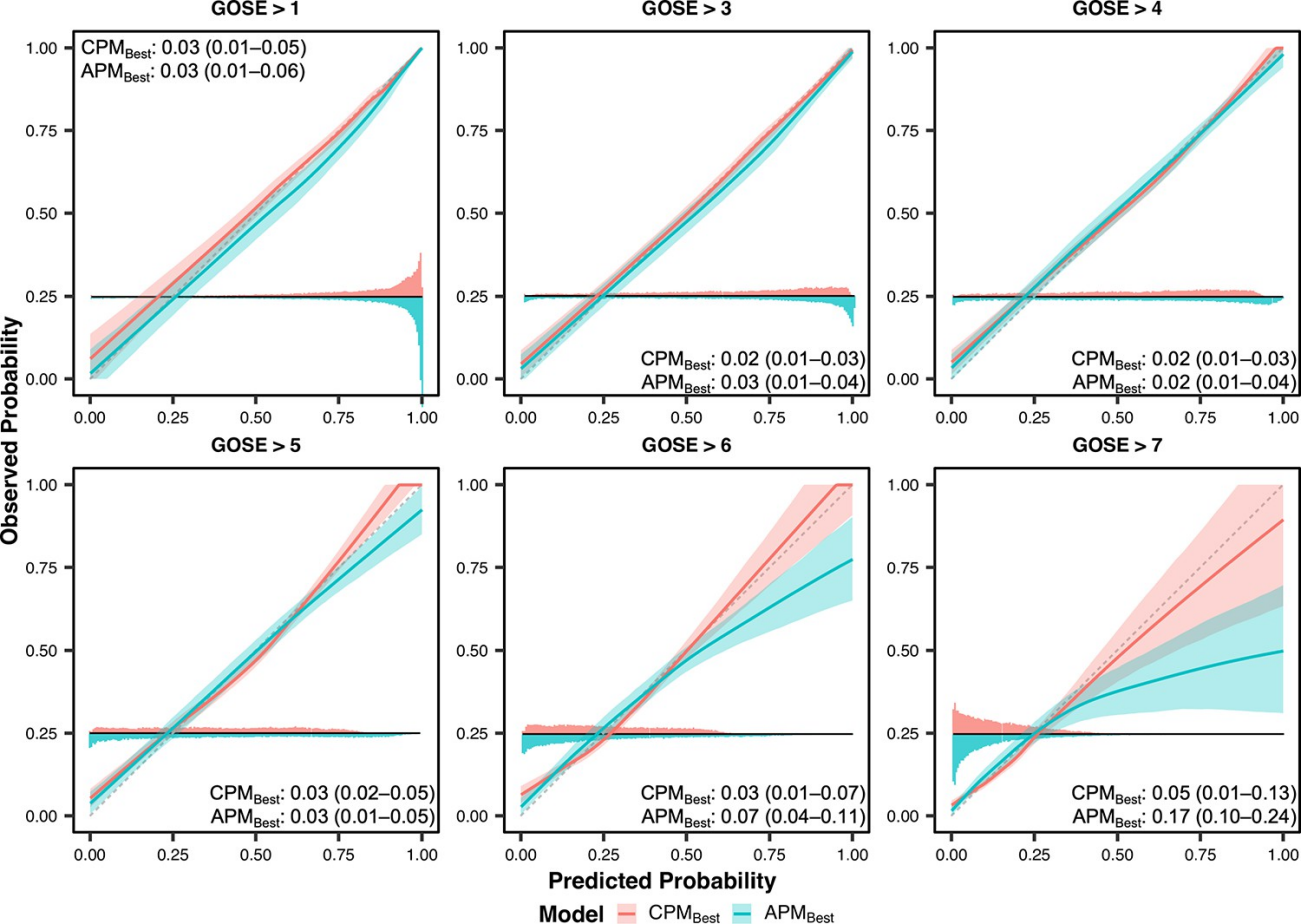
Ordinal calibration curves of best-performing concise-predictor-based model (CPM_Best_) and best-performing all-predictor-based model (APM_Best_). GOSE = Glasgow Outcome Scale–Extended at 6 months post-injury. In each panel, a comparative histogram (200 uniform bins), centred at a horizontal line in the bottom quarter, displays the distribution of predicted probabilities for CPM_Best_ (above the line) and APM_Best_ (below the line) at the given GOSE threshold. CPM_Best_ and APM_Best_ correspond to the CPM (**S2 Table**) and APM (**S3 Table**), respectively, with the lowest unweighted average of integrated calibration indices (ICI) across the thresholds. Shaded areas are 95% confidence intervals derived using bias-corrected bootstrapping (1,000 resamples) to represent the variation across repeated *k*-fold cross-validation folds (20 repeats of 5 folds) and, for CPM_Best_, 100 missing value imputations. The values in each panel correspond to the mean ICI (95% confidence interval) at the given threshold. The diagonal dashed line represents the line of perfect calibration (ICI = 0).

The calibration slopes and calibration curves for the best-calibrated CPMs (CPM_Best_), compared against those for the best-calibrated APMs (APM_Best_), are displayed in **Table 4** and **Fig 3**, respectively. Unlike CPM_Best_, APM_Best_ could not avoid significant overfitting at the three highest GOSE thresholds (i.e., GOSE > 5, GOSE > 6, and GOSE > 7). At these thresholds, we observe that the calibration curve of APM_Best_ significantly veered off the diagonal line of ideal calibration for higher predicted probabilities. However, due to the relative infrequency of these predictions (comparative histograms in **Fig 3**), the ICI of APM_Best_ is not significantly higher than that of CPM_Best_. Our results suggest that APM_Best_ requires more patients with higher functional outcomes, in both the training and validation sets, to mitigate overfitting [45].

### Predictor importance

**Fig 4:**
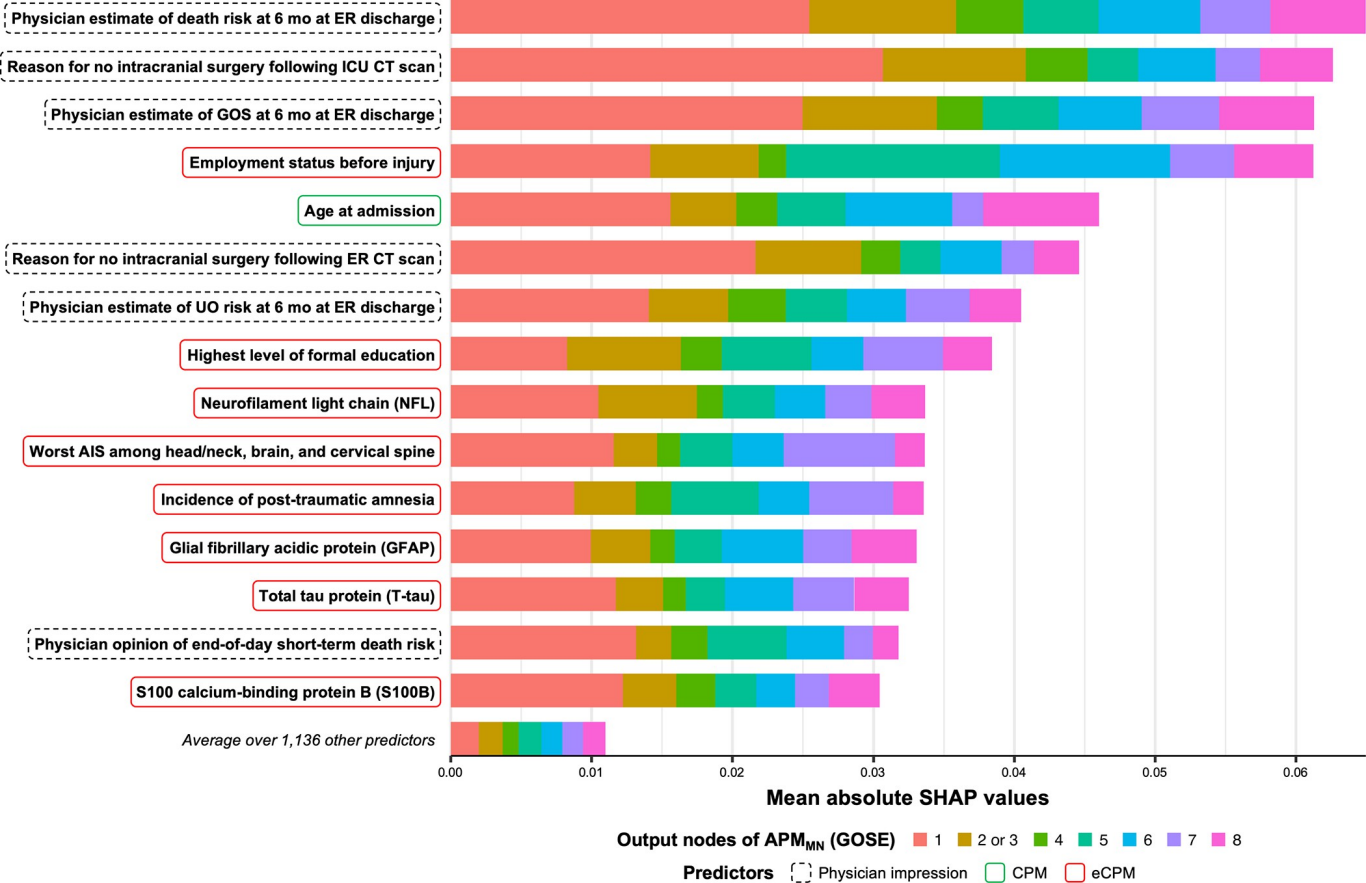
Mean absolute shapley additive explanation (SHAP) values of most important predictors for multinomial-encoding all-predictor-based model (APM_MN_). ICU = intensive care unit. ER = emergency room. CT = computerised tomography. GOS = Glasgow Outcome Scale (not extended). UO = unfavourable outcome, defined by functional dependence (i.e., GOSE ≤ 4). AIS = Abbreviated Injury Scale. GOSE = Glasgow Outcome Scale–Extended at 6 months post-injury. CPM = predictors that are included in the original concise predictor set. eCPM = predictors that are added to the original concise predictor set to form the extended concise predictor set. The mean absolute SHAP value is interpreted as the average magnitude of the relative additive contribution of a predictor’s most important token towards the predicted probability at each GOSE score for a single patient. Predictor types are denoted by the coloured boundary around predictor names. Physician impression predictors denote predictors that encode the explicit impressions or rationales of ICU physicians and are not considered for the extended concise predictor set.

Given that APM_MN_ significantly outperforms APM_OR_ in discrimination and calibration, we focus the assessment of predictor importance to APM_MN_. A bar plot of the mean absolute SHAP values associated with the 15 most important predictors in APM_MN_ is provided in **Fig 4**. We find that the subjective early prognoses of ICU physicians had the greatest contribution towards APM_MN_ predictions, particularly for the prediction of death (GOSE = 1) within 6 months. Initially, this result (along with the high contribution of other physician impressions) seems to suggest that integration of a physician’s interpretations of a patient’s baseline status may add important prognostic information. These impressions likely summarise information from a variable number of other predictors along with the physician’s own experience-based judgement, resulting in high prediction contributions. However, inclusion of these variables may result in problematic self-fulfilling prophecies [51]. For instance, a physician’s poor prognosis directly influences WLSM, which was instituted in 144 (70.2%) of the 205 patients who died in the ICU [52]. Including a variable for physician prognosis may then negatively bias the outcome prediction and unduly promote WLSM. Therefore, we do not consider physician impression predictors for our extended concise predictor set. We also observe that ‘age at admission’ was the only concise predictor among the 15 most important ones. The importance ranks (out of 1,151) of the concise predictors (**Table 2**) are: age = 5^th^, glucose = 23^rd^, Marshall CT = 25^th^, pupillary reactivity = 29^th^, GCSm = 42^nd^, haemoglobin = 50^th^, hypoxia = 284^th^, tSAH = 301^st^, EDH = 414^th^, and hypotension = 420^th^. The eight remaining predictors of the top 15 (**Fig 4**) were added to the concise predictor set to form our extended concise predictor set. Within the tokens for “employment status before injury,” we found that the single token indicating retirement is much more important than the others. Thus, instead of encoding all 10 options for employment status, we included a single indicator variable for retirement in our extended concise predictor set. The eight added predictors included 2 demographic variables (retirement status and highest level of formal education), 4 protein biomarker concentrations (neurofilament light chain [NFL], glial fibrillary acidic protein [GFAP], total tau protein [T-tau], and S100 calcium-binding protein B [S100B]), and 2 clinical assessment variables (worst abbreviated injury score [AIS] among head, neck, brain, and cervical spine injuries and incidence of post-traumatic amnesia at ICU admission). The extended concise predictor set, including the ten original concise predictors and the eight added predictors, is statistically characterised in **S1 Table**.

A bar plot of the mean absolute SHAP values of APM_MN_ for each of the five folds of the first repeat is provided in **S5 Fig**. Most of the eight added predictors, along with age at admission, are consistently represented among the most important predictors across the five folds.

### eCPM discrimination and calibration

The discrimination and calibration metrics for the best-performing extended-predictor-based model (eCPM_Best_) are listed in **Table 4**. Inclusion of the eight selected predictors accounted for about half of the gains in discrimination performance achieved by APM_Best_ over CPM_Best_ according to ORC, Somers’ *D*_*xy*_, and the dichotomous *c*-indices. Based on the difference in Somers’ *D*_*xy*_, the eight added predictors allowed models to explain an additional 6% of the ordinal variation in GOSE at 6 months post-injury. Unlike APM_Best_, eCPM_Best_ is not significantly overfitted at any threshold. The calibration curves of eCPMs (**S6 Fig**) are largely similar to those of the corresponding CPMs (**S3 Fig**), except at the highest threshold (i.e., GOSE > 7). Similar to those of APM_MN_, the calibration curves of eCPMs veer off the line of ideal calibration at higher predicted probabilities of GOSE > 7. The eCPM results support the finding that discrimination performance can be improved with the expansion of the predictor set. Furthermore, by limiting the number of added predictors and the analytical complexity of the model, eCPM avoided the significant miscalibration of APM at higher thresholds.

The discrimination and calibration metrics for each eCPM are listed in **S4 Table**.

## Discussion

To our knowledge, this is the most comprehensive evaluation of early ordinal outcome prognosis for critically ill TBI patients. Our analysis cross-compares a range of ordinal prediction modelling strategies with a large range of available baseline predictors to determine the relative contribution of each towards model performance. Employing an AI tokenisation and embedding technique, we develop highly flexible ordinal prediction models that can learn from the entire, heterogeneous set of 1,151 predictors, available within the first 24 hours of ICU stay, in the CENTER-TBI dataset. This information includes not only all baseline clinical data currently deemed significant for ICU care of TBI but also advanced sub-study results (e.g., protein biomarkers, central haemostatic markers, genetic markers, and advanced MRI results) that represent the experimental frontier of clinical TBI assessment [1, 15, 16]. Therefore, our work reveals the interpretable limits of baseline ordinal, 6-month GOSE prediction in the ICU at this time.

Our key finding is that augmenting the baseline predictor set was much more relevant for improving ordinal model prediction performance than was increasing analytical complexity with deep learning. Within a given predictor set, artificial neural networks did not perform better than logistic regression models (**S2 and S4 Tables**), nor did models with additional hidden layers for the APMs (**S4 Appendix**). This result is consistent with findings in the binary prediction case [53]. On the other hand, augmenting the predictor set, from CPM to APM, substantially improved ordinal discrimination (ORC: +8.6%, **Table 4**) and prediction at lower GOSE thresholds (e.g., GOSE > 1 *c*-index: +8.4%, **Table 4**). Just adding eight predictors to the concise predictor set accounted for about half of the gains in discrimination. However, the addition of predictors negatively affected model calibration, particularly at higher GOSE thresholds (**Fig 3**, **Table 4**). This result underlines the need for careful consideration of probability calibration during model development (e.g., recalibrate with isotonic regression to mitigate overfitting).

At the same time, our results also indicate that ordinal early outcome prognosis for critically ill TBI patients is limited in capability. The best-performing model, which learns from all baseline information in the CENTER-TBI dataset, can only correctly discriminate two randomly chosen patients with two randomly chosen GOSE scores 76% (95% CI: 74%– 77%) of the time. Equivalently, if the best performing model was tasked with ranking seven randomly chosen patients–each with a different true GOSE–by predicted GOSE, an average 5.10 (95% CI: 4.74–5.46) of the 21 possible pairwise orderings will be incorrect. Currently, ordinal model outputs explain, at best, 57% (95% CI: 54%– 60%) of the ordinal variation in 6-month GOSE. Ordinal prediction models struggle to reliably predict full recovery (GOSE > 7 *c*-index: 75% [95% CI: 72%– 79%], **Table 4**), and gains from expanding the predictor set diminish with higher GOSE thresholds.

It is important to acknowledge that the predictor importance results of this article should not be interpreted for predictor discovery or validation. SHAP values are visualised (**Fig 4**) solely to globally interpret APM_MN_ predictions and to form the extended concise predictor set. Risk factor validation, which falls out of the scope of this work, would require investigating the robustness and clinical plausibility of the relationship between predictor values and their corresponding SHAP values [54]. Moreover, causal analysis with apt consideration of confounding factors or dataset biases would be necessary before commenting on the potential effects or mechanisms of individual predictors.

We recognise several limitations in our study. While the concise predictor set was originally designed for prognosis after moderate-to-severe TBI [8] (i.e., baseline GCS 3–12), 26.6% of our study population had experienced mild (i.e., baseline GCS 13–15) TBI (**Table 1**). Predictor sets have been designed for mild TBI patients (e.g., UPFRONT study predictors [55]). However, in line with the aims of the CENTER-TBI project [15], we focus the TBI population not by initial characterisation with GCS but by stratum of care (i.e., admission to the ICU). Therefore, we selected the single concise predictor set that was best validated for the majority of critically ill TBI patients. Our outcome categories (GOSE at 6 months post-injury) were statistically imputed for 13% of our dataset using available GOSE between 2 weeks and one-year post-injury. Although this method was strongly validated on the same (CENTER-TBI) dataset [18], we do recognise that our outcome labels may not be precisely correct. The focus of this work is on the prediction of functional outcomes through GOSE; nonetheless, it is worth considering other outcomes, such as quality-of-life and psychological health, that are important for clinical decision making [56]. Finally, before the AI models developed in this work and in subsequent iterations could be integrated into ICU practice, limitations of generalisability must be addressed [57]. Our models were developed on a multicentre, adult population, prospectively recruited between 2014 and 2017 [25], across Europe, and may encode recruitment, collection, and clinical biases native to our patient set. AI models must continuously be updated, iteratively retrained on incoming information, to help fight the effect these biases may have on returned prognoses for a given patient.

In the setting of TBI prognosis, we encourage the use of AI not to add analytical complexity (i.e., make models “deeper”) but to expand the predictor set (i.e., make models “wider”). Studies have uncovered promising prognostic value in neuro-inflammatory markers [58, 59] and high-resolution TBI monitoring and imaging modalities (e.g., intracranial and cerebral perfusion pressure [60–62], accelerometery [63], and MRI [64–66]), and we recommend integrating these features into ordinal prognostic models, especially to improve prediction of higher functional outcomes. We also believe that there is a feasible performance limit to reliable ordinal outcome prognosis if only statically considering the clinical information from the first 24 hours of ICU stay. It would seem far-fetched to expect all relevant information pertaining to an outcome at 6 months to be encapsulated in the first 24 hours of ICU treatment. Heterogeneous pathophysiological processes unfold over time in patients after TBI [67, 68], and dynamic prediction models, which return model outputs longitudinally with changing clinical information, are better equipped to consider these temporal effects on prognosis. Dynamic prognosis models have been developed for TBI patients [69] and the greater ICU population (not exclusive to TBI) [35, 70, 71], but none of them predict functional outcomes on an ordinal scale. We suggest that the next iteration of this work should be to develop ordinal dynamic prediction models on all clinical information available during the complete ICU stay.

## Ethical approval statement

The CENTER-TBI study has been conducted in accordance with all relevant laws of the European Union and all relevant laws of the country where the recruiting sites were located, including (but not limited to) the relevant privacy and data protection laws and regulations, the relevant laws and regulations on the use of human materials, and all relevant guidance relating to clinical studies from time in force including (but not limited to) the ICH Harmonised Tripartite Guideline for Good Clinical Practice (CPMP/ICH/135/95) and the World Medical Association Declaration of Helsinki entitled “Ethical Principles for Medical Research Involving Human Subjects.” Written informed consent by the patients and/or the legal representative/next of kin was obtained (according to local legislation) for all patients recruited in the core dataset of CENTER-TBI and documented in the electronic case report form. Ethical approval was obtained for each recruiting site.

The list of sites, ethical committees, approval numbers and approval dates can be found on the website: https://www.center-tbi.eu/project/ethical-approval.

## Supporting information

S1 Appendix Explanation of selected ordinal prediction models for CPM and eCPM. (PDF)

S2 Appendix Explanation of APM for ordinal GOSE prediction. (PDF)

S3 Appendix Detailed explanation of ordinal model performance and calibration metrics. (PDF)

S4 Appendix Hyperparameter optimisation results. (PDF)

S1 Fig CONSORT-style flow diagram for patient enrolment and follow-up. CENTER-TBI = Collaborative European NeuroTrauma Effectiveness Research in TBI. ICU = intensive care unit. GOSE = Glasgow Outcome Scale–Extended. MSM = Markov multi-state model (see **Materials and methods**). The dashed, olive-green line in the lower-middle of the diagram divides the enrolment flow diagram (above) and the follow-up breakdown (below).
(TIF)

S2 Fig Characterisation of missingness among concise predictor set. U.P. = unreactive pupils. GCSm = motor component score of the Glasgow Coma Scale. Hb = haemoglobin. Glu. = glucose. HoTN = hypotension. Marshall = Marshall computerised tomography classification. tSAH = traumatic subarachnoid haemorrhage. EDH = extradural haematoma. (**A**) Proportion of total sample size (*n* = 1,550) with missing values for each IMPACT extended model predictor. (**B**) Missingness matrix where each column represents a concise predictor, and each row represents a combination of missing predictors (red) and non-missing predictors (blue) found in the dataset. The prevalence of each combination (i.e., row) in the study population is shown with a horizontal histogram (far right) labelled with the proportion of the study population with the corresponding combination of missing predictors. For example, the bottom row of the matrix shows that 54.77% of the study population had no missing concise predictors while the penultimate row shows that 14.71% of the study population had only glucose and haemoglobin missing among the concise predictors.
(TIF)

S3 Fig Ordinal calibration curves of each concise-predictor-based model (CPM). GOSE = Glasgow Outcome Scale–Extended at 6 months post-injury. Shaded areas are 95% confidence intervals derived using bias-corrected bootstrapping (1,000 resamples) to represent the variation across repeated *k*-fold cross-validation folds (20 repeats of 5 folds) and 100 missing value imputations. The values in each panel correspond to the mean integrated calibration index (ICI) (95% confidence interval) at the given threshold. The diagonal dashed line represents the line of perfect calibration (ICI = 0). The CPM types (CPM_MNLR_, CPM_POLR_, CPM_DeepMN_, and CPM_DeepOR_) are decoded in the **Materials and methods** and described in **S1 Appendix**.
(TIF)

S4 Fig Ordinal calibration curves of each all-predictor-based model (APM). GOSE = Glasgow Outcome Scale–Extended at 6 months post-injury. Shaded areas are 95% confidence intervals derived using bias-corrected bootstrapping (1,000 resamples) to represent the variation across repeated *k*-fold cross-validation folds (20 repeats of 5 folds). The values in each panel correspond to the mean integrated calibration index (ICI) (95% confidence interval) at the given threshold. The diagonal dashed line represents the line of perfect calibration (ICI = 0). The APM types (APM_MN_ and APM_OR_) are decoded in the **Materials and methods** and described in **S2 Appendix**.
(TIF)

S5 Fig Mean absolute SHAP values of the most important predictors for APM_MN_ in each of the five folds of the first repeat. ICU = intensive care unit. CT = computerised tomography. ER = emergency room. GOS = Glasgow Outcome Scale (not extended). AIS = Abbreviated Injury Scale. UO = unfavourable outcome, defined by functional dependence (i.e., GOSE ≤ 4). FIBTEM = fibrin-based extrinsically activated test with tissue factor and cytochalasin D. GOSE = Glasgow Outcome Scale–Extended at 6 months post-injury. The mean absolute SHAP value is interpreted as the average magnitude of the relative additive contribution of a predictor’s most important token towards the predicted probability at each GOSE score for a single patient.
(TIF)

S6 Fig Ordinal calibration curves of each extended concise-predictor-based model (eCPM). GOSE = Glasgow Outcome Scale–Extended at 6 months post-injury. Shaded areas are 95% confidence intervals derived using bias-corrected bootstrapping (1,000 resamples) to represent the variation across repeated *k*-fold cross-validation folds (20 repeats of 5 folds) and 100 missing value imputations. The values in each panel correspond to the mean integrated calibration index (ICI) (95% confidence interval) at the given threshold. The diagonal dashed line represents the line of perfect calibration (ICI = 0). The eCPM types (eCPM_MNLR_, eCPM_POLR_, eCPM_DeepMN_, and eCPM_DeepOR_) are decoded in the **Materials and methods** and described in **S1 Appendix**.
(TIF)

S1 Table Extended concise baseline predictors of the study population stratified by ordinal 6-month outcomes. (PDF)

S2 Table Ordinal concise-predictor-based model (CPM) discrimination and calibration performance. (PDF)

S3 Table Ordinal all-predictor-based model (APM) discrimination and calibration performance. (PDF)

S4 Table Ordinal extended concise-predictor-based model (eCPM) discrimination and calibration performance. (PDF)
