# Supplementary material for: The leap to ordinal: Detailed functional prognosis after traumatic brain injury with a flexible modelling approach
Source: PLoS One. 2022 Jul 5;17(7):e0270973. doi: 10.1371/journal.pone.0270973 (PMC9255749; doi:10.1371/journal.pone.0270973)
Supplement: S1 Appendix — (PDF) [file pone.0270973.s001.pdf]

S1 Appendix: Explanation of selected ordinal prediction models for CPM and eCPM

Multinomial logistic regression (MNLR)

CPM_{MNLR} and $eCPM_{MNLR}$ were implemented using the 'MNLogit' class from the 'statsmodels' module (dev. v0.14.0) [1] in Python (v3.7.6). The GOSE score of 1 (death) was designated as the reference label, and, for each other GOSE score, a separate logistic model was trained to regress the logit of the ratio of the probability of that score to the reference score from a linear combination of the predictors. The logit outputs of each model feed into a softmax function, after which cumulative sums would determine the probability at each threshold. Model weights for MNLR were optimised using the Broyden–Fletcher–Goldfarb–Shanno (BFGS) algorithm [2] to maximize conditional likelihood.

Proportional odds (i.e., ordinal) logistic regression (POLR)

CPM_{POLR} and $eCPM_{POLR}$ were implemented using the 'OrderedModel' class from the 'statsmodels' module in Python. The model maps GOSE scores to a latent, logit space where consecutive GOSE scores are separated by thresholds. Thus, the model trains only one set of linear predictor weights, but a separate intercept for each threshold. Model weights for POLR were optimised using the Broyden–Fletcher–Goldfarb–Shanno (BFGS) algorithm [2] to maximize conditional likelihood.

Class-weighted feedforward neural network with a multinomial output layer (DeepMN)

CPM_{DeepMN} and $eCPM_{DeepMN}$ were implemented using the 'PyTorch' (v1.10.0) [3] module in Python. The network architecture of DeepMN included a hyperparametric number of dense hidden layers (either 1, 2, 3, 4, 5, or 6), each containing a hyperparametric number of nodes (either 128, 256, or 512) with a rectified linear unit (ReLU) activation function and a hyperparametric percentage (either 0% or 20%) dropout during training. The output layer of DeepMN was a softmax layer of 7 nodes, from which probabilities at each GOSE are calculated with cumulative sums (**Fig 1A**). DeepMN was optimised using the Adam algorithm (γ [learning rate] = 0.001, β_1 = 0.9, β_2 = 0.999) [4] with categorical cross-entropy loss. In the loss function, classes were weighted inversely proportional to the frequency of each GOSE score in the training set to counter class imbalance.

Class-weighted feedforward neural network with an ordinal output layer (DeepOR)

CPM_{DeepOR} and $eCPM_{DeepOR}$ were implemented using the 'PyTorch' (v1.10.0) [3] module in Python. The network architecture of DeepMN included a hyperparametric number of

dense hidden layers (either 1, 2, 3, 4, 5, or 6), each containing a hyperparametric number of nodes (either 128, 256, or 512) with a rectified linear unit (ReLU) activation function and a hyperparametric percentage (either 0% or 20%) dropout during training. The output layer of DeepOR was a sigmoid layer of 6 nodes, where each node represented the binomial probability of the outcome being greater than a certain threshold, and each node is constrained to be less than or equal to lower-threshold nodes with a negative ReLU transformation (**Fig 1A**). DeepOR was optimised using the Adam algorithm (γ [learning rate] = 0.001, β_1 = 0.9, β_2 = 0.999) with binary cross-entropy loss. In the loss function, classes were weighted inversely proportional to the frequency of each GOSE score in the training set to counter class imbalance.

**CPM or eCPM**	**Description**	**Hyperparameters**			**Total number of configurations**
		**Hidden layers**	**Neurons per layer***	**Dropout**	
MNLR	Multinomial logistic regression				1
POLR	Proportional odds (i.e., ordinal) logistic regression				1
DeepMN	Class-weighted feedforward neural network with a multinomial (i.e., softmax) output layer	1, 2, 3, 4, 5, or 6	128, 256, or 512	0% or 20%	2184
DeepOR	Class-weighted feedforward neural network with an ordinal (i.e., sigmoid at each threshold) output layer	1, 2, 3, 4, 5, or 6	128, 256, or 512	0% or 20%	2184

*Different hidden layers may have distinct numbers of neurons.

References

1. Seabold S, Perktold J. Statsmodels: Econometric and Statistical Modeling with Python. In: van der Walt S, Millman J, editors. Proceedings of the 9th Python in Science Conference (SciPy 2010). Austin: SciPy; 2010. pp. 92-96. doi: 10.25080/Majora-92bf1922-011
2. Fletcher R. Practical Methods of Optimization. 2nd ed. New York: John Wiley & Sons; 1987.
3. Paszke A, Gross S, Massa F, Lerer A, Bradbury J, Chanan G, et al. PyTorch: An Imperative Style, High-Performance Deep Learning Library. In: Wallach H, Larochelle H, Beygelzimer A, d'Alché-Buc F, Fox E, Garnett R, editors. Advances in Neural Information Processing Systems 32 (NeurIPS 2019). Vancouver: NeurIPS; 2019.
4. Kingma DP, Ba J. Adam: A Method for Stochastic Optimization. arXiv:1412.6980v9 [Preprint]. 2017 [cited 2021 December 26]. Available from: https://arxiv.org/abs/1412.6980
