# Supplementary material for: The leap to ordinal: Detailed functional prognosis after traumatic brain injury with a flexible modelling approach
Source: PLoS One. 2022 Jul 5;17(7):e0270973. doi: 10.1371/journal.pone.0270973 (PMC9255749; doi:10.1371/journal.pone.0270973)
Supplement: S2 Appendix — (PDF) [file pone.0270973.s002.pdf]

S2 Appendix: Explanation of APM for ordinal GOSE prediction

APM_{MN} and APM_{OR} were implemented using the 'PyTorch' (v1.10.0) [1] module in Python. Regarding hyperparameters, the embedding and weight-averaging layer (**Fig 2B**) is considered to the be the first hidden layer. Thus, the number of neurons for the first hidden layer can also be considered as the embedding dimension (i.e., the length of each of the embedding vectors trained on the token dictionary). The individual vector returned by the embedding and weight-averaging layer (**Fig 2B**) then undergoes a hyperparametric number of dense hidden layers (either 0, 1, 2, 3, 4, or 5), each containing a hyperparametric number of nodes (either 128, 256, or 512) with a rectified linear unit (ReLU) activation function and a hyperparametric percentage (either 0% or 20%) dropout during training. The output layer of APM_{MN} was a softmax layer of 7 nodes, from which probabilities at each GOSE are calculated with cumulative sums (**Fig 1A**). APM_{MN} was optimised using the Adam algorithm (γ [learning rate] = 0.001, β_1 = 0.9, β_2 = 0.999) [2] with categorical cross-entropy loss. In the loss function, classes were weighted inversely proportional to the frequency of each GOSE score in the training set to counter class imbalance. The output layer of APM_{OR} was a sigmoid layer of 6 nodes, where each node represented the binomial probability of the outcome being greater than a certain threshold, and each node is constrained to be less than or equal to lower-threshold nodes with a negative ReLU transformation (**Fig 1A**). APM_{OR} was optimised using the Adam algorithm (γ [learning rate] = 0.001, β_1 = 0.9, β_2 = 0.999) with binary cross-entropy loss. In the loss function, classes were weighted inversely proportional to the frequency of each GOSE score in the training set to counter class imbalance.

**APM**	**Description**	**Hyperparameters**			**Total number of configurations**
		**Hidden layers***	**Neurons per layer†**	**Dropout**	
APM_{MN}	Class-weighted embedding and weight-averaging layer followed by a feedforward neural network with a multinomial (i.e., softmax) output layer	1, 2, 3, 4, 5, or 6	128, 256, or 512	0% or 20%	2184
APM_{OR}	Class-weighted embedding and weight-averaging layer followed by a feedforward neural network with an ordinal (i.e., sigmoid at each threshold) output layer	1, 2, 3, 4, 5, or 6	128, 256, or 512	0% or 20%	2184

*The first hidden layer corresponds to the embedding and weight-averaging layer.
†Different hidden layers may have distinct numbers of neurons.

References

1. Paszke A, Gross S, Massa F, Lerer A, Bradbury J, Chanan G, et al. PyTorch: An Imperative Style, High-Performance Deep Learning Library. In: Wallach H, Larochelle H, Beygelzimer A, d'Alché-Buc F, Fox E, Garnett R, editors. Advances in Neural Information Processing Systems 32 (NeurIPS 2019). Vancouver: NeurIPS; 2019.

2. Kingma DP, Ba J. Adam: A Method for Stochastic Optimization. arXiv:1412.6980v9 [Preprint]. 2017 [cited 2021 December 26]. Available from: https://arxiv.org/abs/1412.6980
