# Supplementary material for: The leap to ordinal: Detailed functional prognosis after traumatic brain injury with a flexible modelling approach
Source: PLoS One. 2022 Jul 5;17(7):e0270973. doi: 10.1371/journal.pone.0270973 (PMC9255749; doi:10.1371/journal.pone.0270973)
Supplement: S3 Appendix — (PDF) [file pone.0270973.s003.pdf]

S3 Appendix: Detailed explanation of ordinal model performance and calibration metrics

In this appendix, we will describe each of our selected testing set discrimination, classification, and calibration metrics in mathematical and interpretive detail. Much of this information has already been published by Van Calster et al [1] and Austin et al [2], but we summarise and adapt it here for the ease of the reader. For each of the metrics, we derive the no information value (NIV), which corresponds to the metric value a model would theoretically achieve in the absence of predictive information, and the ideal, full information value (FIV).

Discrimination performance metrics

First, as a reference, let us define the dichotomous *c*-index, also known as the area under the receiver operating characteristic curve (AUC). Let us first assume a dichotomous prediction problem, in which there are N_1 patients with outcome 1 and N_2 patients with outcome 2. For a patient of outcome 1, let us denote the predicted probability of outcome 1 as p_{1,n_1}, where $n_1 \in [\![1, N_1]\!]$. Likewise, for a patient of outcome 2, let us denote the predicted probability of outcome 1 as p_{1,n_2}, where $n_2 \in [\![1, N_2]\!]$. The dichotomous *c*-index is then defined as:

$$c = \frac{1}{N_1 N_2} \sum_{n_1=1}^{N_1} \sum_{n_2=1}^{N_2} I_{p_{1,n_1} > p_{1,n_2}}$$

where $I_{p_{1,n_1} > p_{1,n_2}}$ is an indicator variable defined by:

$$I_{p_{1,n_1} > p_{1,n_2}} = \begin{cases} 1 \text{ if } p_{1,n_1} > p_{1,n_2}; \\ 0.5 \text{ if } p_{1,n_1} = p_{1,n_2}; \\ 0 \text{ otherwise.} \end{cases}$$

Thus, the dichotomous *c*-index can be interpreted as the probability that a model correctly separates 2 patients of different outcome. The dichotomous *c*-index is the most widely used discrimination metric for binary outcome prediction; however, there is no trivial extension for ordinal outcome prediction [3]. In this appendix, we explore the extensions used for our study.

Ordinal *c*-index (ORC)

The ordinal *c*-index (ORC), developed by Van Calster et al [1], is the primary metric of model discrimination performance in our study. Consider a set of 7 randomly chosen patients, each of one of the GOSE scores in our study, such that each patient is represented by n_o where $o \in \{1{,}2 \text{ or } 3, 4{,}5{,}6{,}7{,}8\}$. Now suppose an ordinal GOSE prediction model, such as one of those presented in **Fig 1A**, receives this set of patients

and is tasked with ranking the patients in order of predicted functional outcome. Let $\Pr^{(n_o)}(GOSE > t)$ represent the predicted probability, returned by our model, at threshold $t \in \{1,3,4,5,6,7\}$ for patient $n_o \in \{n_1, n_{2\text{ or }3}, n_4, n_5, n_6, n_7, n_8\}$ in our set. One way the model could achieve this ranking is to start with the lowest threshold ($GOSE > 1$), select the patient with the lowest probability at this threshold (i.e., $\underset{n_o}{\operatorname{argmin}} \Pr^{(n_o)}(GOSE > 1)$), set that patient aside as the lowest ranked patient, move on to the subsequent threshold ($GOSE > 3$), repeat this process for the remaining patients, and repeat at subsequent thresholds until a single patient remains for the highest rank. The ideal predicted ranking would be $n_1 < n_{2\text{ or }3} < n_4 < n_5 < n_6 < n_7 < n_8$. The primary rationale behind ORC is to calculate the average proportional "closeness" between the model-predicted ranking and this ideal ranking. To achieve a mathematical definition for closeness, the developers of ORC considered a scenario: suppose the model-predicted ranking of the given set is: $n_1 < n_4 < n_5 < n_{2\text{ or }3} < n_6 < n_8 < n_7$. From this predicted ranking, we would require at least 3 pairwise switching steps to achieve the target rank. For example:

- *Step 1*: switch n_4 and $n_{2\text{ or }3}$. *Result*: $n_1 < n_{2\text{ or }3} < n_5 < n_4 < n_6 < n_8 < n_7$
- *Step 2*: switch n_5 and n_4. *Result*: $n_1 < n_{2\text{ or }3} < n_4 < n_5 < n_6 < n_8 < n_7$
- *Step 3*: switch n_8 and n_7. *Result*: $n_1 < n_{2\text{ or }3} < n_4 < n_5 < n_6 < n_7 < n_8$

Let us define *S* as the number of necessary pairwise switching steps (i.e., the number of incorrect pairwise orderings) to reach the ideal ranking. Trivially, the ideal *S* ($S_{\min}$) is 0. In the worst possible scenario, in which the predicted ranking is a complete reversal of the ideal ranking (i.e., $n_8 < n_7 < n_6 < n_5 < n_4 < n_{2\text{ or }3} < n_1$), one would require the maximum number of unique pairwise switching steps possible to achieve the ideal ranking. Since we have 7 possible outcome categories, this is equivalent to $S_{max} = \binom{7}{2} = 21$. In the case of a tie, we add 0.5 to *S*. The definition of the proportion of closeness, denoted as C, between the model-predicted ranking and the ideal ranking for a given set is thus:

$$C = 1 - \frac{S}{S_{max}} = 1 - \frac{S}{21}$$

In the example provided above, where $S = 3$, the proportional closeness between the predicted ranking and the ideal ranking is $C = 1 - \frac{3}{21} \approx 0.86$. Thus, to define ORC as the average proportional closeness in ranking over all possible sets,

$$\boldsymbol{ORC} = \frac{1}{N_1 N_{2\text{ or }3} N_4 N_5 N_6 N_7 N_8} \sum_{n_1=1}^{N_1} \sum_{n_{2\text{ or }3}=1}^{N_{2\text{ or }3}} \sum_{n_4=1}^{N_4} \sum_{n_5=1}^{N_5} \sum_{n_6=1}^{N_6} \sum_{n_7=1}^{N_7} \sum_{n_8=1}^{N_8} C_{n_1, n_{2\text{ or }3}, n_4, n_5, n_6, n_7, n_8}$$

where $N_o \; \forall \; o \in \{1, 2 \text{ or } 3, 4,5,6,7,8\}$ denotes the number of patients of GOSE score o, and $C_{n_1 n_{2\text{ or }3} n_4 n_5 n_6 n_7 n_8}$ denotes the proportional closeness of the model ranking to the ideal ranking for patient set $\{n_1, n_{2\text{ or }3}, n_4, n_5, n_6, n_7, n_8\}$. Furthermore, if we simplify this formula:

$$ORC = \frac{1}{N_1 N_{2\text{ or }3} N_4 N_5 N_6 N_7 N_8} \sum_{n_1=1}^{N_1} \sum_{n_{2\text{ or }3}=1}^{N_{2\text{ or }3}} \sum_{n_4=1}^{N_4} \sum_{n_5=1}^{N_5} \sum_{n_6=1}^{N_6} \sum_{n_7=1}^{N_7} \sum_{n_8=1}^{N_8} C_{n_1,n_{2\text{ or }3},n_4,n_5,n_6,n_7,n_8}$$

$$= \frac{1}{N_1 N_{2\text{ or }3} N_4 N_5 N_6 N_7 N_8} \sum_{n_1=1}^{N_1} \sum_{n_{2\text{ or }3}=1}^{N_{2\text{ or }3}} \sum_{n_4=1}^{N_4} \sum_{n_5=1}^{N_5} \sum_{n_6=1}^{N_6} \sum_{n_7=1}^{N_7} \sum_{n_8=1}^{N_8} \left[1 - \frac{S_{n_1,n_{2\text{ or }3},n_4,n_5,n_6,n_7,n_8}}{S_{max}}\right]$$

$$= \frac{1}{N_1 N_{2\text{ or }3} N_4 N_5 N_6 N_7 N_8} \sum_{n_1=1}^{N_1} \sum_{n_{2\text{ or }3}=1}^{N_{2\text{ or }3}} \sum_{n_4=1}^{N_4} \sum_{n_5=1}^{N_5} \sum_{n_6=1}^{N_6} \sum_{n_7=1}^{N_7} \sum_{n_8=1}^{N_8} \left[\frac{S_{max} - S_{n_1,n_{2\text{ or }3},n_4,n_5,n_6,n_7,n_8}}{S_{max}}\right]$$

$$= \frac{1}{N_1 N_{2\text{ or }3} N_4 N_5 N_6 N_7 N_8} \sum_{n_1=1}^{N_1} \sum_{n_{2\text{ or }3}=1}^{N_{2\text{ or }3}} \sum_{n_4=1}^{N_4} \sum_{n_5=1}^{N_5} \sum_{n_6=1}^{N_6} \sum_{n_7=1}^{N_7} \sum_{n_8=1}^{N_8} \left[\frac{1}{\binom{7}{2}} \sum_{i=1}^{6} \sum_{j=i+1}^{7} (S_{max} - S_{n_1,n_{2\text{ or }3},n_4,n_5,n_6,n_7,n_8})\right]$$

$$= \frac{1}{N_1 N_{2\text{ or }3} N_4 N_5 N_6 N_7 N_8} \sum_{n_1=1}^{N_1} \sum_{n_{2\text{ or }3}=1}^{N_{2\text{ or }3}} \sum_{n_4=1}^{N_4} \sum_{n_5=1}^{N_5} \sum_{n_6=1}^{N_6} \sum_{n_7=1}^{N_7} \sum_{n_8=1}^{N_8} \left[\frac{1}{\binom{7}{2}} \sum_{i=1}^{7} \sum_{j=i+1}^{8} I_{o_{n_j} > o_{n_i}}\right]$$

$$= \frac{1}{\binom{7}{2}} \sum_{i=1}^{7} \sum_{j=i+1}^{8} \left[\frac{1}{N_1 N_{2\text{ or }3} N_4 N_5 N_6 N_7 N_8} \sum_{n_1=1}^{N_1} \sum_{n_{2\text{ or }3}=1}^{N_{2\text{ or }3}} \sum_{n_4=1}^{N_4} \sum_{n_5=1}^{N_5} \sum_{n_6=1}^{N_6} \sum_{n_7=1}^{N_7} \sum_{n_8=1}^{N_8} I_{o_{n_j} > o_{n_i}}\right]$$

$$= \frac{1}{\binom{7}{2}} \sum_{i=1}^{7} \sum_{j=i+1}^{8} \left[\frac{1}{N_i N_j} \sum_{n_i=1}^{N_i} \sum_{n_j=1}^{N_j} I_{o_{n_j} > o_{n_i}}\right]$$

$$\boxed{= \frac{1}{\binom{7}{2}} \sum_{i=1}^{7} \sum_{j=i+1}^{8} c_{ij}}$$

which is equivalent to the unweighted average of all pairwise *c*-indices. Therefore, another interpretation of ORC is the probability of a model correctly separating 2 randomly selected patients of 2 randomly selected GOSE scores. Moreover, since the NIV of the *c*-index is 0.5 for random guessing and the FIV is 1, we know that ORC shares the same feasible range of values: $\mathbf{NIV_{ORC}}$ = 0.5 and $\mathbf{FIV_{ORC}}$ = 1. Finally, if there were only 2 possible ordinal outcome categories, we observe that ORC collapses into the dichotomous *c*-index.

The ORC is independent of the prevalence of each GOSE score in the dataset, as each possible set of patients is equally weighted regardless of frequency.

Somers' D_{xy}

The generalised *c*-index, described by Harrell et al [4,5], is defined as the proportion of possible pairs of patients of different functional outcomes in the entire study population which the model correctly discriminates. A pair of patients of different outcomes is defined as a comparable pair and a pair of patients of different outcomes that is correctly discriminated is defined as a concordant pair. Let N^{comp} denote the total number of comparable pairs in the study set and let N^{conc} denote the total number of concordant pairs in the study set. Thus, the generalised *c*-index is defined as:

$$\text{Generalised } c - \text{index} = \frac{N^{conc}}{N^{comp}}$$

Upon simplification,

$$= \frac{N^{conc}}{\sum_{i=1}^{7}\sum_{j=i+1}^{8} N_i N_j}$$

$$= \frac{\sum_{i=1}^{7}\sum_{j=i+1}^{8} N_{ij}^{conc}}{\sum_{i=1}^{7}\sum_{j=i+1}^{8} N_i N_j}$$

$$= \frac{\sum_{i=1}^{7}\sum_{j=i+1}^{8} N_i N_j c_{ij}}{\sum_{i=1}^{7}\sum_{j=i+1}^{8} N_i N_j}$$

we find that the generalised *c*-index is equivalent to a prevalence-weighted average of pairwise *c*-indices. Therefore, the generalised *c*-index shares the same feasible range of values as the dichotomous *c*-index: $\text{NIV}_{\text{Generalised } c\text{-index}} = 0.5$ and $\text{FIV}_{\text{Generalised } c\text{-index}} = 1$. However, in contrast to ORC, generalised *c*-index is dependent on the prevalence of GOSE scores in the patient set.

Somers' D_{xy} [6,7] is defined as the proportion of the difference between the number of concordant pairs and the number of discordant pairs to the total number of comparable pairs:

$$\textbf{Somers' } \boldsymbol{D_{xy}} = \frac{N^{conc} - N^{discord}}{N^{comp}}$$

Upon simplification,

$$= \frac{N^{conc} - (N^{comp} - N^{conc})}{N^{comp}}$$

$$= \frac{2N^{conc} - N^{comp}}{N^{comp}}$$

$$= 2\frac{N^{conc}}{N^{comp}} - 1$$

$$= 2(\text{Generalised } c - \text{index}) - 1$$

we observe the relationship between Somers' D_{xy} and the generalised *c*-index. Therefore, the feasible range of Somers' D_{xy} is: **NIV$_{\text{Somers' } D_{xy}}$** = 2(0.5) – 1 = 0 and **FIV$_{\text{Somers' } D_{xy}}$** = 2(1) – 1 = 1. Moreover, Somers' D_{xy} is also dependent on the prevalence of GOSE scores in the patient set. Somers' D_{xy} can also be interpreted as the proportion of ordinal variation in the outcome that can be explained by the variation in model output.

Threshold-level dichotomous *c*-index

The threshold-level dichotomous *c*-indices represent the probability of the model correctly discriminating 2 randomly selected patients, one on each side of the threshold of functional recovery. The average of the threshold-level *c*-indices across the 6 possible GOSE thresholds represents the probability of the model correctly discriminating 2 patients, one on each side of a randomly selected GOSE threshold. The average threshold-level dichotomous *c*-index is also a prevalence-weighted form of the pairwise *c*-index, though weighting is not perfectly aligned with prevalence as with the generalised *c*-index [1]. The feasible range of dichotomous *c*-indices are: **NIV$_{\text{Dichotomous } c\text{-index}}$** = 0.5 to **FIV$_{\text{Dichotomous } c\text{-index}}$** = 1.

Probability calibration metrics

Threshold-level calibration slope

Let $Y \in \{0,1\}$ designate the true outcome at a threshold of GOSE and let $p_{pred} \in [0,1]$ designate the predicted probability value returned by a model at this threshold. The logistic recalibration framework [8] fits the following model from the testing set predictions: $\text{logit}(Y) = \beta_0 + \beta_1 \text{logit}(p_{pred})$. β_1 represents the calibration slope [9]. When $\beta_0 = 0$ and $\beta_1 = 1$, the model is calibrated. When $\beta_1 < 1$, the model is overfitted and returns too extreme values: higher p_{pred} are overestimated while lower p_{pred} are underestimated. When $\beta_1 > 1$, the model is underfitted and the converse is true. We do not focus on β_0 in our study because, in the setting of cross-validation, β_0 is not relevant [10].

Threshold-level Integrated calibration index (ICI)

On the threshold-level probabilities and threshold-level outcomes of the testing set predictions, we fit a locally weighted scatterplot smoothing (LOWESS) function [11] to return the observed probability at each predicted probability value [12]. The range of corresponding observed probability for each predicted probability is visualised in a

smoothed probability calibration plot (**Fig 3B**). Let $p_{pred} \in [0,1]$ denote a predicted probability value and $p_{obs}(p_{pred}) \in [0,1]$ denote the corresponding observed probability value. Then, the calibration error function, denoted as $E_{calibration}$, is defined as: $\boldsymbol{E_{calibration}(p_{pred})} = |p_{obs}(p_{pred}) - p_{pred}|$.

The integrated calibration index (ICI) corresponds to the mean calibration error [2]. Since the ideal calibration error is 0, the $\mathbf{FIV_{ICI}}$ is trivially 0. However, the calculation of the NIV varies based on the outcome distribution at each threshold.

Consider the case of random guessing during prediction at a given threshold. This implies that the model returns predicted probabilities uniformly from 0 to 1, regardless of any patient information (**S3A.1 Fig**). Therefore, the corresponding observed probability at each predicted probability value equals π_{above}, the proportion of patients above the given threshold (**S3A.1 Fig**). In other words, there is no association between predicted and observed probabilities, and the model is completely uncalibrated.

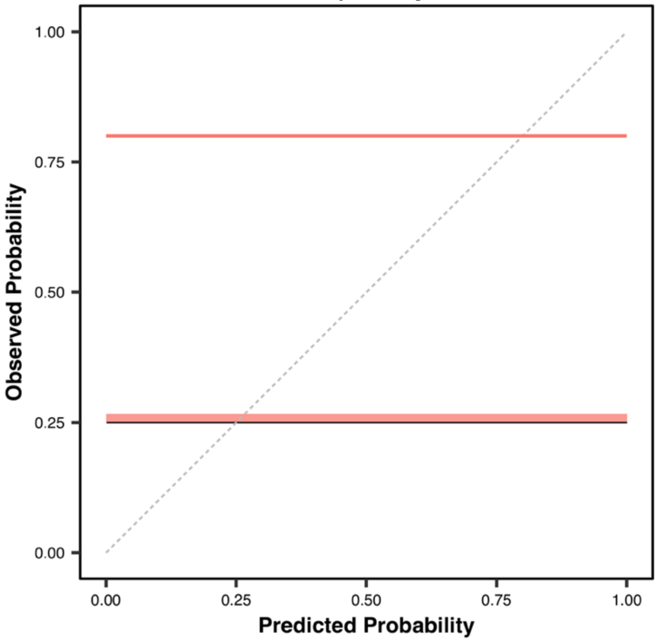

S3A.1 Fig. Example of a probability calibration curve for a random-guessing prediction model at a given threshold of GOSE. The histogram (200 uniform bins), centred at the horizontal line in the bottom quarter, displays the uniform distribution of predicted probabilities for a random guessing model. This plot assumes that the proportion of patients above the threshold (π_{above}) is 0.8.

From the probability calibration curve (**S3A.1 Fig**), we derive a graphical representation of the probability density function of $E_{calibration}$ in **S3A.2 Fig**. This corresponds to an asymmetrical (if $\pi_{above} \neq 0.5$) distribution with density 2 up to $E_{calibration} = \min\{\pi_{above}, 1 - \pi_{above}\}$ and then density 1 from $E_{calibration} = \min\{\pi_{above}, 1 - \pi_{above}\}$ to $E_{calibration} = \max\{\pi_{above}, 1 - \pi_{above}\}$ (**S3A.2 Fig**).

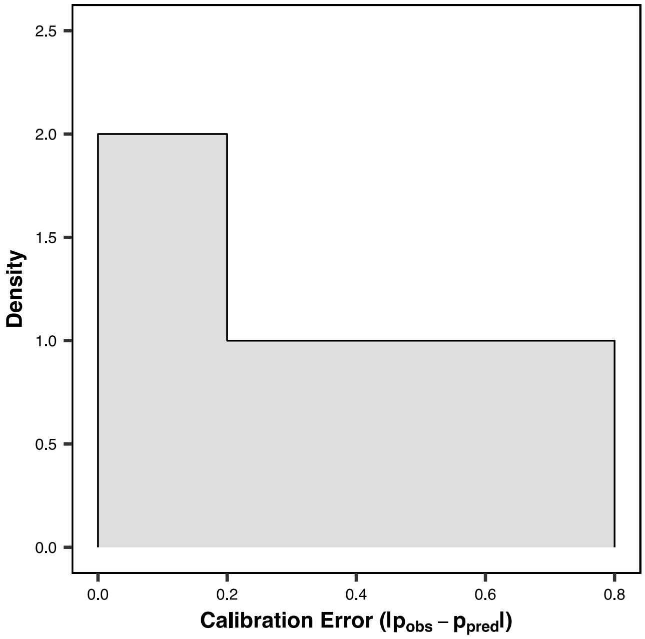

S3A.2 Fig. Example of probability density of calibration error for a random-guessing prediction model at a given threshold of GOSE. This plot assumes that the proportion of patients above the threshold (π_{above}) is 0.8.

ICI is equivalent to the integral of the calibration error function over all returned probability prediction values:

$$\boldsymbol{ICI} = \frac{1}{\max\{p_{pred}\} - \min\{p_{pred}\}} \int_{\min\{p_{pred}\}}^{\max\{p_{pred}\}} f_{P_{pred}}(p_{pred})\, E_{calibration}(p_{pred})\, dp_{pred}$$

where $f_{P_{pred}}(p_{pred})$ represents the probability density function over p_{pred}values. For the random-guessing model, we determined that p_{obs} is constant, i.e., $p_{obs}(p_{pred}) = \pi_{above}\ \forall\ p_{pred} \in [0,1]$ at each threshold. Moreover, p_{pred} is distributed uniformly from 0 to 1. Therefore:

$$\boldsymbol{NIV_{ICI}} = \int_0^1 E_{calibration}(p_{pred})\, dp_{pred}$$

$$= \int_0^1 \left|\pi_{above} - p_{pred}\right| dp_{pred}$$

$$= \int_0^{\pi_{above}} (\pi_{above} - p_{pred})\, dp_{pred} + \int_{\pi_{above}}^1 (p_{pred} - \pi_{above})\, dp_{pred}$$

$$= \frac{1}{2}\pi_{above}^2 + \frac{1}{2}(1 - \pi_{above})^2$$

$$= \pi_{above}^2 - \pi_{above} + \frac{1}{2}$$

A graphical representation of cumulative distribution up to the NIV_{ICI} for our example is provided in **S3A.3 Fig**.

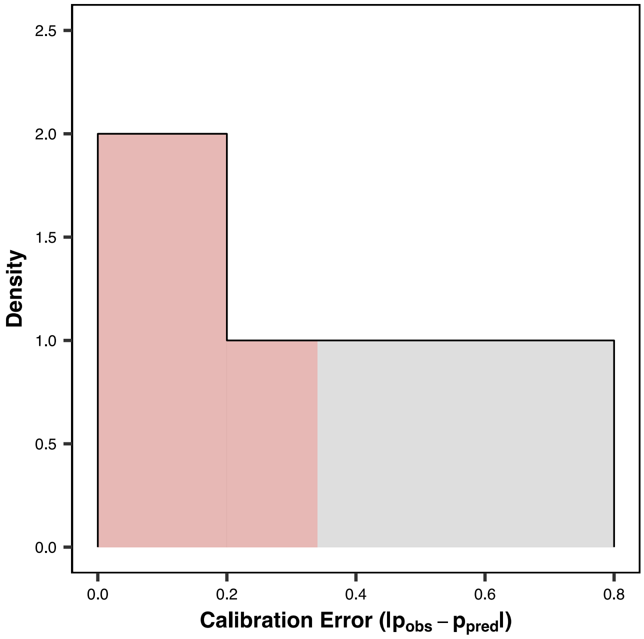

S3A.3 Fig. Example of cumulative probability density up to ICI for a random-guessing prediction model at a given threshold of GOSE. This plot assumes that the proportion of patients above the threshold (π_{above}) is 0.8. The ICI equals 0.34 in calibration error.

References

1. Van Calster B, Van Belle V, Vergouwe Y, Steyerberg EW. Discrimination ability of prediction models for ordinal outcomes: Relationships between existing measures and a new measure. Biom J. 2012;54: 674-685. doi: 10.1002/bimj.201200026.
2. Austin PC, Steyerberg EW. The Integrated Calibration Index (ICI) and related metrics for quantifying the calibration of logistic regression models. Statistics in Medicine. 2019;38: 4051-4065. doi: 10.1002/sim.8281.
3. Hilden J. The Area under the ROC Curve and Its Competitors. Med Decis Making. 1991;11: 95-101. doi: 10.1177/0272989X9101100204.
4. Harrell FE, Califf RM, Pryor DB, Lee KL, Rosati RA. Evaluating the Yield of Medical Tests. JAMA. 1982;247: 2543-2546. doi: 10.1001/jama.1982.03320430047030.
5. Harrell FE. Regression Modeling Strategies. 2nd ed. Cham: Springer; 2015. doi: 10.1007/978-3-319-19425-7.
6. Somers RH. A New Asymmetric Measure of Association for Ordinal Variables. Am Sociol Rev. 1962;27: 799-811. doi: 10.2307/2090408.
7. Kim J. Predictive Measures of Ordinal Association. Am J Sociol. 1971;76: 891-907. doi: 10.1086/225004.

8. Cox DR. Two further applications of a model for binary regression. Biometrika. 1958;45: 562-565. doi: 10.1093/biomet/45.3-4.562.
9. Miller ME, Langefeld CD, Tierney WM, Hui SL, McDonald CJ. Validation of Probabilistic Predictions. Med Decis Making. 1993;13: 49-57. doi: 10.1177/0272989X9301300107.
10. Van Calster B, Nieboer D, Vergouwe Y, De Cock B, Pencina MJ, Steyerberg EW. A calibration hierarchy for risk models was defined: from utopia to empirical data. J Clin Epidemiol. 2016;74: 167-176. doi: 10.1016/j.jclinepi.2015.12.005.
11. Cleveland WS. Robust Locally Weighted Regression and Smoothing Scatterplots. J Am Stat Assoc. 1979;74: 829-836. doi: 10.1080/01621459.1979.10481038.
12. Austin PC, Steyerberg EW. Graphical assessment of internal and external calibration of logistic regression models by using loess smoothers. Stat Med. 2014;33: 517-535. doi: 10.1002/sim.5941.
