# Supplementary material for: The leap to ordinal: Detailed functional prognosis after traumatic brain injury with a flexible modelling approach
Source: PLoS One. 2022 Jul 5;17(7):e0270973. doi: 10.1371/journal.pone.0270973 (PMC9255749; doi:10.1371/journal.pone.0270973)
Supplement: S4 Appendix — (PDF) [file pone.0270973.s004.pdf]

S4 Appendix: Hyperparameter optimisation results

Training for each of the parametric models (CPM_{DeepMN}, CPM_{DeepOR}, APM_{MN}, APM_{OR}, $eCPM_{DeepMN}$, and $eCPM_{DeepOR}$) was made more efficient by dropping out consistently underperforming parametric configurations, on the validation sets, with the Bootstrap Bias Corrected with Dropping Cross-Validation (BBCD-CV) method [1]. During configuration dropout, the optimal configuration for each model was determined over all existing validation set predictions up to that point, and 1,000 resamples of unique patients were drawn to form bootstrapping resamples for the testing of suboptimal configurations versus the optimal configuration in terms of ordinal *c*-index (ORC) [2]. If a given suboptimal configuration was unable to match or outperform the optimal configuration in at least 5% of the resamples, it was dropped out from training in future repeated *k*-fold cross-validation partitions.

Each of the models began repeated *k*-fold cross-validation training with 2,184 parametric configurations (as detailed in **S1 Appendix** and **S2 Appendix**). Under the repeated *k*-fold cross validation scheme of our study, models were trained in the order of repeats (from 1 to 20), and, within each repeat, in the order of folds (from 1 to 5). After training all viable configurations up to a certain partition, BBCD-CV was performed. The decision of which partitions was dependent on the number of remaining viable configurations and the availability of relevant cores (e.g., APM training required GPUs) on the high-performance computer (HPC), and thus varied by model. Since models of the same predictor set were trained together (i.e., CPM_{DeepMN} and CPM_{DeepOR}), BBCD-CV was performed for each of the models of a certain predictor set at after the same partition and a different optimal configuration was determined for each model.

In this appendix, we demonstrate the results of BBCD-CV hyperparameter optimisation by model type. First, we list the partitions after which BBCD-CV was performed, demonstrate the number of configurations dropped at these points, and characterise the variable hyperparameter distribution of the remaining viable configurations.

Concise-predictor-based models (CPMs)

BBCD-CV was performed thrice for CPM_{DeepMN} and CPM_{DeepOR}, after the end of: (1) repeat 1, (2) repeat 3, and (3) repeat 15. The number of remaining viable configurations after these dropouts is visualised, on a binary logarithmic scale, in **S4A.1 Fig**. The distribution of hyperparameters in the viable configurations, after each dropout, are listed in **S4A.1 Table** and **S4A.2 Table** for CPM_{DeepMN} and CPM_{DeepOR}, respectively.

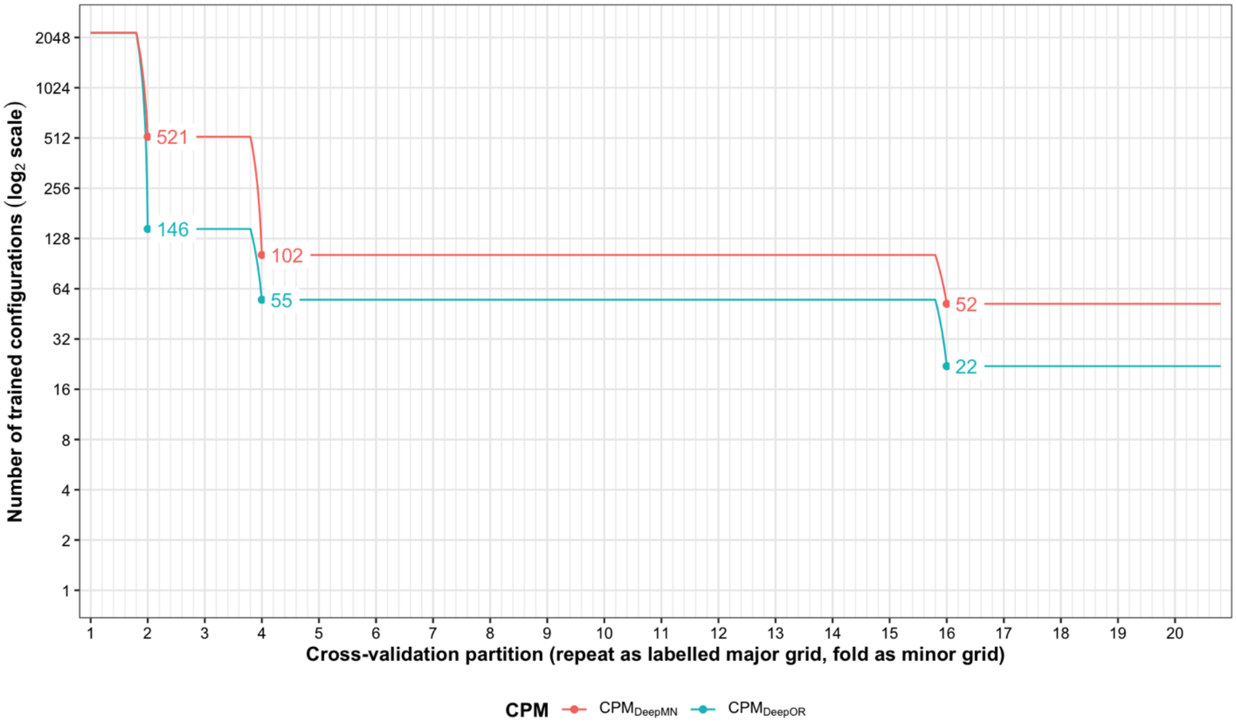

S4A.1 Fig. Number of trained viable configurations for each CPM during repeated *k*-fold cross-validation.

S4A.1 Table. Variable hyperparameter distributions after each dropout for CPM_{DeepMN}.

**Hyperparameter**	**Value**	**Starting configurations** (*n* = 2184)	**Remaining configurations after**		
			**Repeat 1** (*n* = 521)	**Repeat 3** (*n* = 102)	**Repeat 15** (*n* = 52)
Training dropout per layer					
	0	1092 (50.0%)	221 (42.4%)	19 (18.6%)	8 (15.4%)
	0.2	1092 (50.0%)	300 (57.6%)	83 (81.4%)	44 (84.6%)
Number of layers					
	1	6 (0.3%)	0 (0%)	0 (0%)	0 (0%)
	2	18 (0.8%)	3 (0.6%)	2 (2.0%)	1 (1.9%)
	3	54 (2.5%)	10 (1.9%)	4 (3.9%)	4 (7.7%)
	4	162 (7.4%)	32 (6.1%)	12 (11.8%)	8 (15.4%)
	5	486 (22.3%)	143 (27.4%)	57 (55.9%)	38 (73.1%)
	6	1458 (66.8%)	333 (63.9%)	27 (26.5%)	1 (1.9%)
Median number of neurons per layer					
	128	284 (13.0%)	90 (17.3%)	32 (31.4%)	18 (34.6%)
	192	320 (14.7%)	67 (12.9%)	8 (7.8%)	3 (5.8%)
	256	920 (42.1%)	230 (44.1%)	44 (43.1%)	25 (48.1%)
	320	56 (2.6%)	9 (1.7%)	2 (2.0%)	0 (0%)
	384	320 (14.7%)	58 (11.1%)	5 (4.9%)	2 (3.8%)
	512	284 (13.0%)	67 (12.9%)	11 (10.8%)	4 (7.7%)

S4A.2 Table. Variable hyperparameter distributions after each dropout for CPM_{DeepOR}.

**Hyperparameter**	**Value**	**Starting configurations** (*n* = 2184)	**Remaining configurations after**		
			**Repeat 1** (*n* = 146)	**Repeat 3** (*n* = 55)	**Repeat 15** (*n* = 22)

Training dropout per layer					
	0	1092 (50.0%)	42 (28.8%)	13 (23.6%)	5 (22.7%)
	0.2	1092 (50.0%)	104 (71.2%)	42 (76.4%)	17 (77.3%)
Number of layers					
	1	6 (0.3%)	0 (0%)	0 (0%)	0 (0%)
	2	18 (0.8%)	0 (0%)	0 (0%)	0 (0%)
	3	54 (2.5%)	2 (1.4%)	1 (1.8%)	1 (4.5%)
	4	162 (7.4%)	0 (0%)	0 (0%)	0 (0%)
	5	486 (22.3%)	56 (38.4%)	23 (41.8%)	12 (54.5%)
	6	1458 (66.8%)	88 (60.3%)	31 (56.4%)	9 (40.9%)
Median number of neurons per layer					
	128	284 (13.0%)	23 (15.8%)	7 (12.7%)	2 (9.1%)
	192	320 (14.7%)	16 (11.0%)	5 (9.1%)	2 (9.1%)
	256	920 (42.1%)	73 (50.0%)	28 (50.9%)	14 (63.6%)
	320	56 (2.6%)	1 (0.7%)	0 (0%)	0 (0%)
	384	320 (14.7%)	17 (11.6%)	6 (10.9%)	1 (4.5%)
	512	284 (13.0%)	16 (11.0%)	9 (16.4%)	3 (13.6%)

All-predictor-based models (APMs)

BBCD-CV was performed twice for APM_{MN} and APM_{OR}, after the end of: (1) the first fold of repeat 1, and (2) repeat 10. The number of remaining viable configurations after these dropouts is visualised, on a binary logarithmic scale, in **S4A.2 Fig**. The distribution of hyperparameters in the viable configurations, after each dropout, are listed in **S4A.3 Table** and **S4A.4 Table** for APM_{MN} and APM_{OR}, respectively.

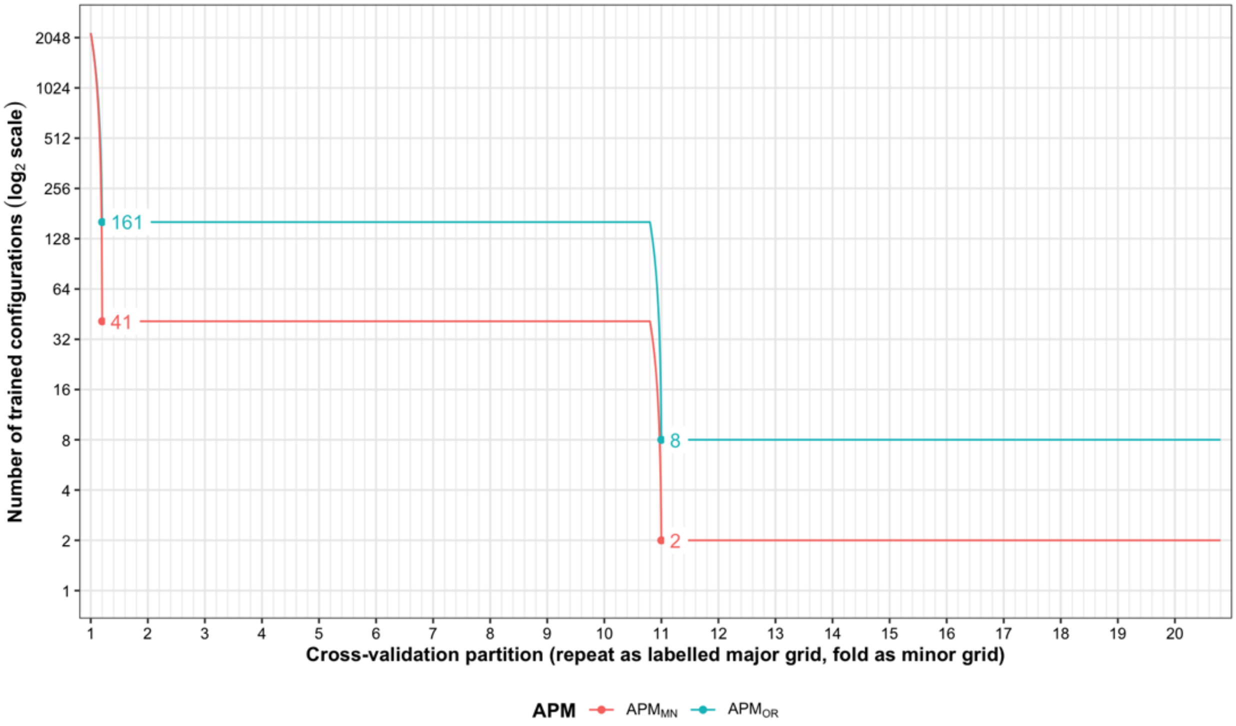

S4A.2 Fig. Number of trained viable configurations for each APM during repeated *k*-fold cross-validation.

S4A.3 Table. Variable hyperparameter distributions after each dropout for APM_{MN}.

Hyperparameter	Value	Starting configurations (*n* = 2184)	Remaining configurations after	
			**Repeat 1, Fold 1** (*n* = 41)	**Repeat 10** (*n* = 2)
Training dropout per layer				
	0	1092 (50.0%)	18 (43.9%)	1 (50.0%)
	0.2	1092 (50.0%)	23 (56.1%)	1 (50.0%)
Number of layers				
	1	6 (0.3%)	3 (7.3%)	2 (100.0%)
	2	18 (0.8%)	2 (4.9%)	0 (0%)
	3	54 (2.5%)	1 (2.4%)	0 (0%)
	4	162 (7.4%)	5 (12.2%)	0 (0%)
	5	486 (22.3%)	5 (12.2%)	0 (0%)
	6	1458 (66.8%)	25 (61.0%)	0 (0%)
Median number of neurons per layer				
	128	284 (13.0%)	3 (7.3%)	0 (0%)
	192	320 (14.7%)	5 (12.2%)	0 (0%)
	256	920 (42.1%)	19 (46.3%)	1 (50.0%)
	320	56 (2.6%)	0 (0%)	0 (0%)
	384	320 (14.7%)	8 (19.5%)	0 (0%)
	512	284 (13.0%)	6 (14.6%)	1 (50.0%)

S4A.4 Table. Variable hyperparameter distributions after each dropout for APM_{OR}.

Hyperparameter	Value	Starting configurations (*n* = 2184)	Remaining configurations after	
			**Repeat 1, Fold 1** (*n* = 161)	**Repeat 10** (*n* = 8)
Training dropout per layer				
	0	1092 (50.0%)	22 (13.7%)	0 (0%)
	0.2	1092 (50.0%)	139 (86.3%)	8 (100.0%)
Number of layers				
	1	6 (0.3%)	1 (0.6%)	0 (0%)
	2	18 (0.8%)	1 (0.6%)	0 (0%)
	3	54 (2.5%)	5 (3.1%)	0 (0%)
	4	162 (7.4%)	13 (8.1%)	1 (12.5%)
	5	486 (22.3%)	36 (22.4%)	2 (25.0%)
	6	1458 (66.8%)	105 (65.2%)	5 (62.5%)
Median number of neurons per layer				
	128	284 (13.0%)	31 (19.3%)	2 (25.0%)
	192	320 (14.7%)	29 (18.0%)	4 (50.0%)
	256	920 (42.1%)	73 (45.3%)	1 (12.5%)
	320	56 (2.6%)	6 (3.7%)	0 (0%)
	384	320 (14.7%)	11 (6.8%)	0 (0%)
	512	284 (13.0%)	11 (6.8%)	1 (12.5%)

Extended concise-predictor-based models (eCPMs)

BBCD-CV was performed twice for $eCPM_{DeepMN}$ and $eCPM_{DeepOR}$, after the end of: (1) repeat 1, and (2) repeat 16. The number of remaining viable configurations after these dropouts is visualised, on a binary logarithmic scale, in **S4A.3 Fig**. The distribution of hyperparameters in the viable configurations, after each dropout, are listed in **S4A.5 Table** and **S4A.6 Table** for $eCPM_{DeepMN}$ and $eCPM_{DeepOR}$, respectively.

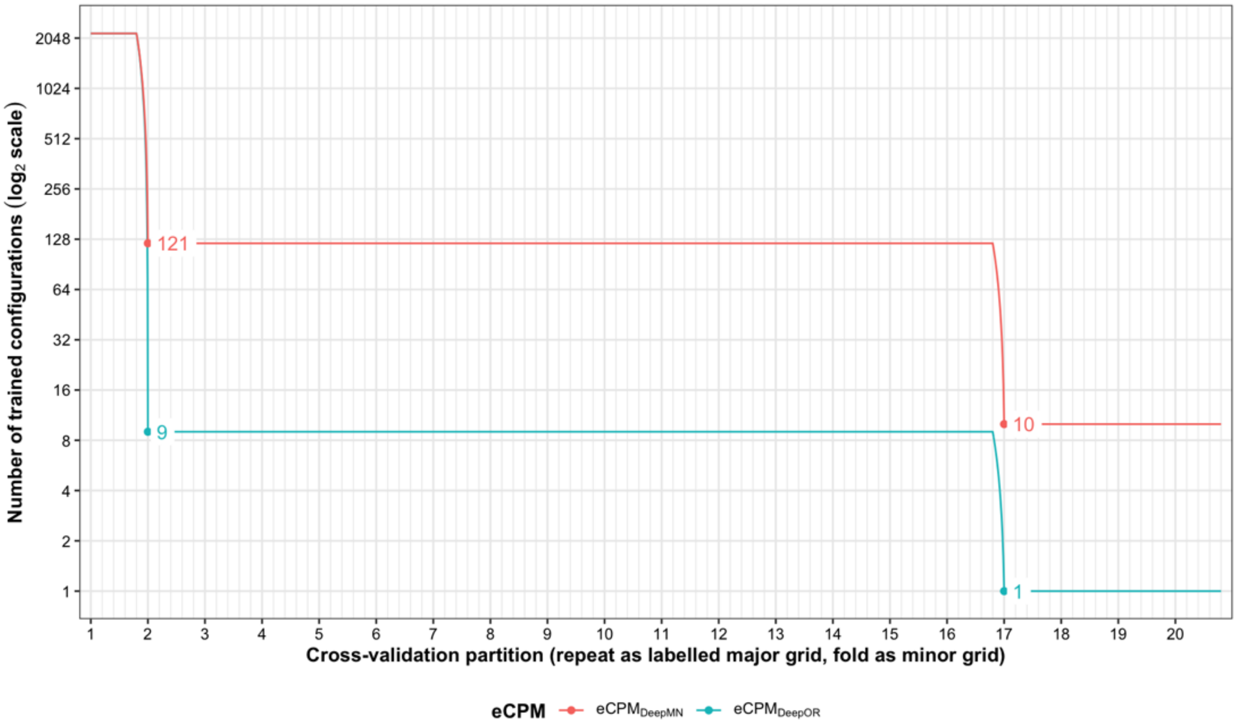

S4A.3 Fig. Number of trained viable configurations for each eCPM during repeated *k*-fold cross-validation.

S4A.5 Table. Variable hyperparameter distributions after each dropout for $eCPM_{DeepMN}$.

**Hyperparameter**	**Value**	**Starting configurations** (n = 2184)	**Remaining configurations after**	
			**Repeat 1** (n = 121)	**Repeat 16** (n = 10)
Training dropout per layer				
	0	1092 (50.0%)	51 (42.1%)	4 (40.0%)
	0.2	1092 (50.0%)	70 (57.9%)	6 (60.0%)
Number of layers				
	1	6 (0.3%)	3 (2.5%)	2 (20.0%)
	2	18 (0.8%)	8 (6.6%)	3 (30.0%)
	3	54 (2.5%)	15 (12.4%)	3 (30.0%)
	4	162 (7.4%)	45 (37.2%)	2 (20.0%)
	5	486 (22.3%)	48 (39.7%)	0 (0%)
	6	1458 (66.8%)	2 (1.7%)	0 (0%)
Median number of neurons per layer				
	128	284 (13.0%)	21 (17.4%)	3 (30.0%)
	192	320 (14.7%)	14 (11.6%)	2 (20.0%)
	256	920 (42.1%)	55 (45.5%)	4 (40.0%)

	320	56 (2.6%)	5 (4.1%)	0 (0%)
	384	320 (14.7%)	11 (9.1%)	0 (0%)
	512	284 (13.0%)	15 (12.4%)	1 (10.0%)

S4A.6 Table. Variable hyperparameter distributions after each dropout for $eCPM_{DeepOR}$.

Hyperparameter	Value	Starting configurations (*n* = 2184)	Remaining configurations after	
			**Repeat 1** (*n* = 9)	**Repeat 16** (*n* = 1)
Training dropout per layer				
	0	1092 (50.0%)	1 (11.1%)	0 (0%)
	0.2	1092 (50.0%)	8 (88.9%)	1 (100.0%)
Number of layers				
	1	6 (0.3%)	1 (11.1%)	1 (100.0%)
	2	18 (0.8%)	4 (44.4%)	0 (0%)
	3	54 (2.5%)	2 (22.2%)	0 (0%)
	4	162 (7.4%)	1 (11.1%)	0 (0%)
	5	486 (22.3%)	0 (0%)	0 (0%)
	6	1458 (66.8%)	1 (11.1%)	0 (0%)
Median number of neurons per layer				
	128	284 (13.0%)	3 (33.3%)	0 (0%)
	192	320 (14.7%)	2 (22.2%)	0 (0%)
	256	920 (42.1%)	3 (33.3%)	1 (100.0%)
	320	56 (2.6%)	1 (11.1%)	0 (0%)
	384	320 (14.7%)	0 (0%)	0 (0%)
	512	284 (13.0%)	0 (0%)	0 (0%)

References

1. Tsamardinos I, Greasidou E, Borboudakis G. Bootstrapping the out-of-sample predictions for efficient and accurate cross-validation. Mach Learning. 2018;107: 1895-1922. doi: 10.1007/s10994-018-5714-4.
2. Van Calster B, Van Belle V, Vergouwe Y, Steyerberg EW. Discrimination ability of prediction models for ordinal outcomes: Relationships between existing measures and a new measure. Biom J. 2012;54: 674-685. doi: 10.1002/bimj.201200026.
