# Supplementary figures and images for: The leap to ordinal: Detailed functional prognosis after traumatic brain injury with a flexible modelling approach
Source: PLoS One. 2022 Jul 5;17(7):e0270973. doi: 10.1371/journal.pone.0270973 (PMC9255749; doi:10.1371/journal.pone.0270973)

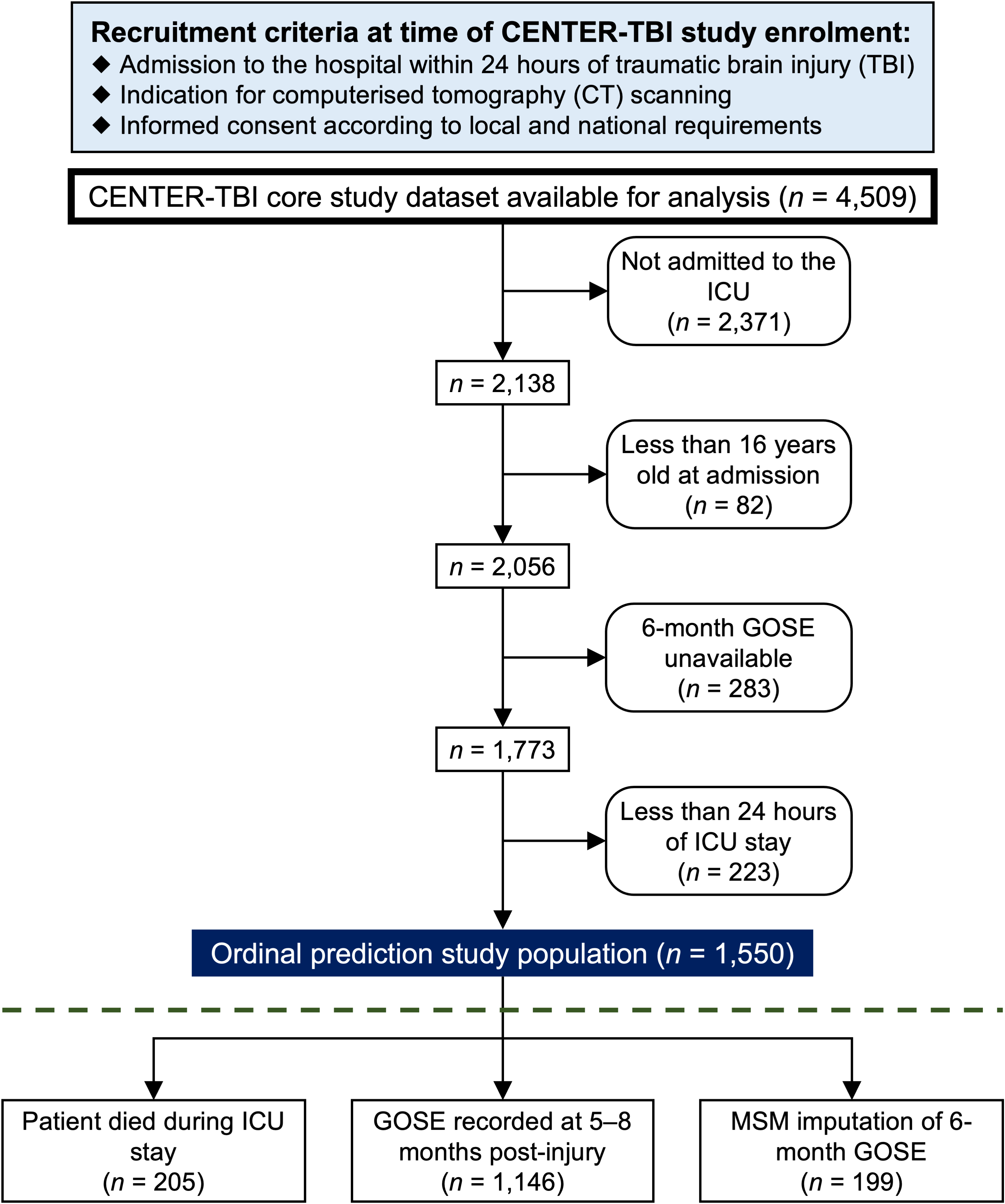

Supplement: S1 Fig — CENTER-TBI = Collaborative European NeuroTrauma Effectiveness Research in TBI. ICU = intensive care unit. GOSE = Glasgow Outcome Scale–Extended. MSM = Markov multi-state model (see Materials and methods). The dashed, olive-green line in the lower-middle of the diagram divides the enrolment flow diagram (above) and the follow-up breakdown (below). (TIF) [file pone.0270973.s005.tif]

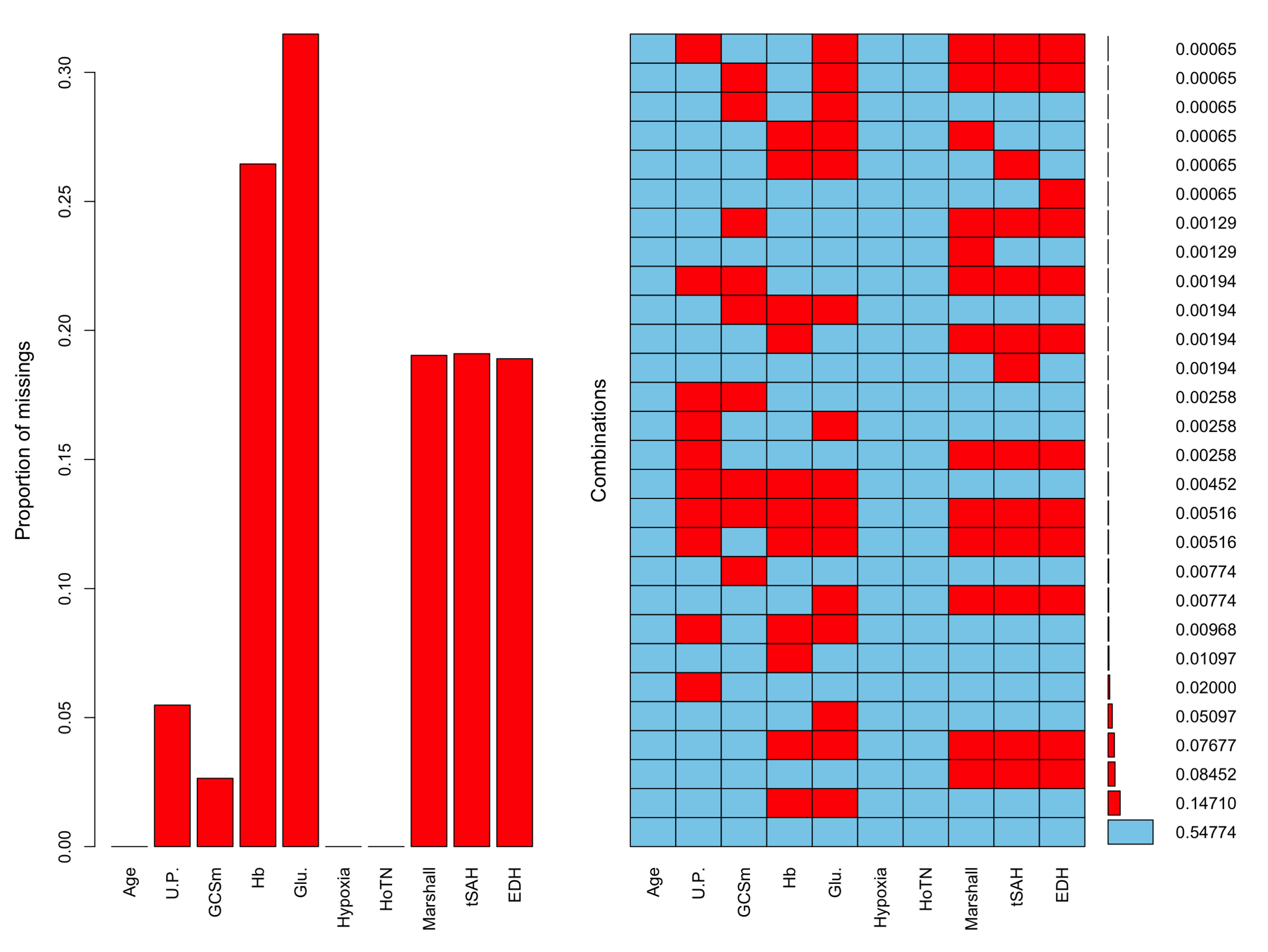

Supplement: S2 Fig — U.P. = unreactive pupils. GCSm = motor component score of the Glasgow Coma Scale. Hb = haemoglobin. Glu. = glucose. HoTN = hypotension. Marshall = Marshall computerised tomography classification. tSAH = traumatic subarachnoid haemorrhage. EDH = extradural haematoma. (A) Proportion of total sample size (n = 1,550) with missing values for each IMPACT extended model predictor. (B) Missingness matrix where each column represents a concise predictor, and each row represents a combination of missing predictors (red) and non-missing predictors (blue) found in the dataset. The prevalence of each combination (i.e., row) in the study population is shown with a horizontal histogram (far right) labelled with the proportion of the study population with the corresponding combination of missing predictors. For example, the bottom row of the matrix shows that 54.77% of the study population had no missing concise predictors while the penultimate row shows that 14.71% of the study population had only glucose and haemoglobin missing among the concise predictors. (TIF) [file pone.0270973.s006.tif]

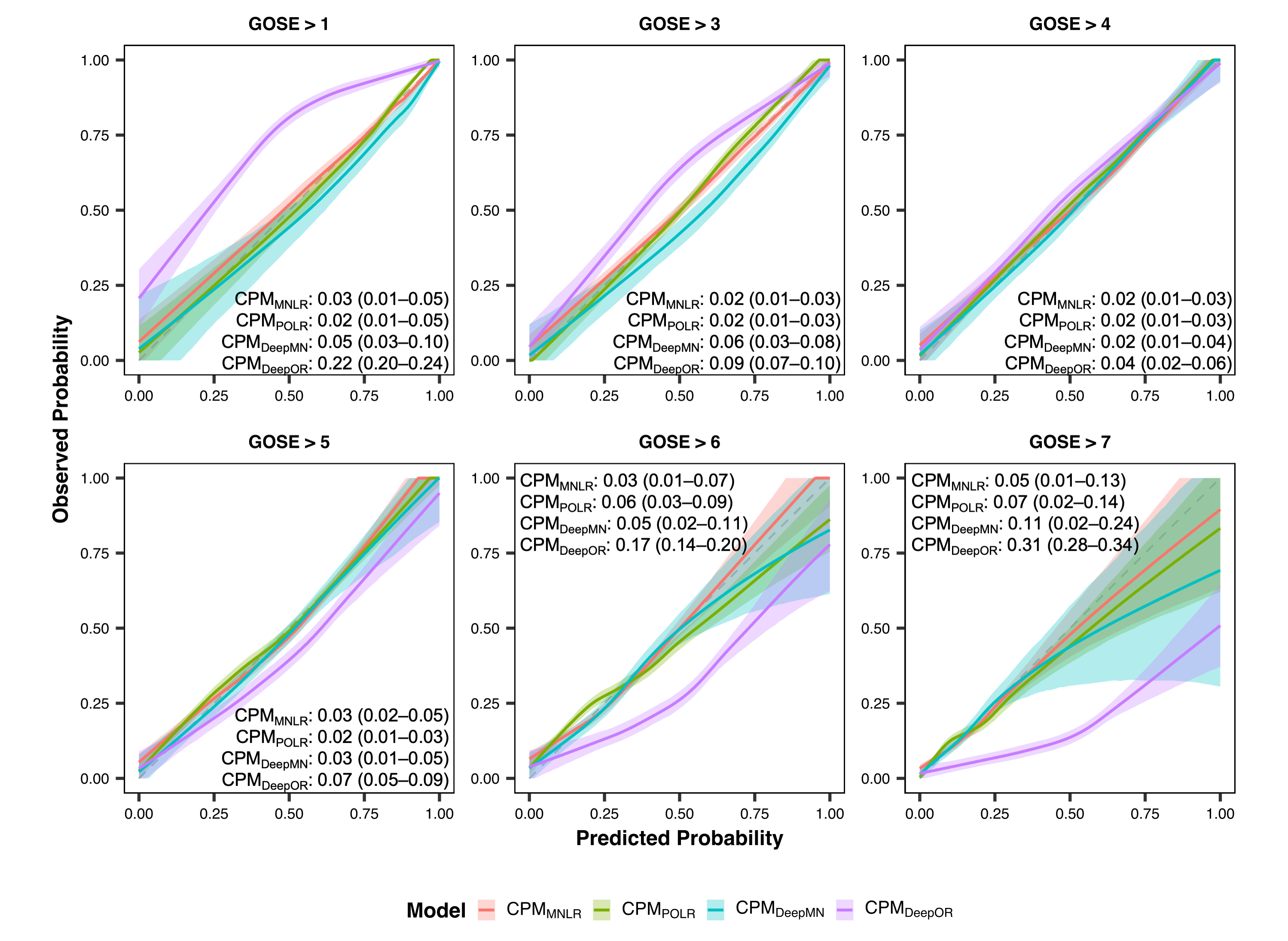

Supplement: S3 Fig — GOSE = Glasgow Outcome Scale–Extended at 6 months post-injury. Shaded areas are 95% confidence intervals derived using bias-corrected bootstrapping (1,000 resamples) to represent the variation across repeated k-fold cross-validation folds (20 repeats of 5 folds) and 100 missing value imputations. The values in each panel correspond to the mean integrated calibration index (ICI) (95% confidence interval) at the given threshold. The diagonal dashed line represents the line of perfect calibration (ICI = 0). The CPM types (CPMMNLR, CPMPOLR, CPMDeepMN, and CPMDeepOR) are decoded in the Materials and methods and described in S1 Appendix. (TIF) [file pone.0270973.s007.tif]

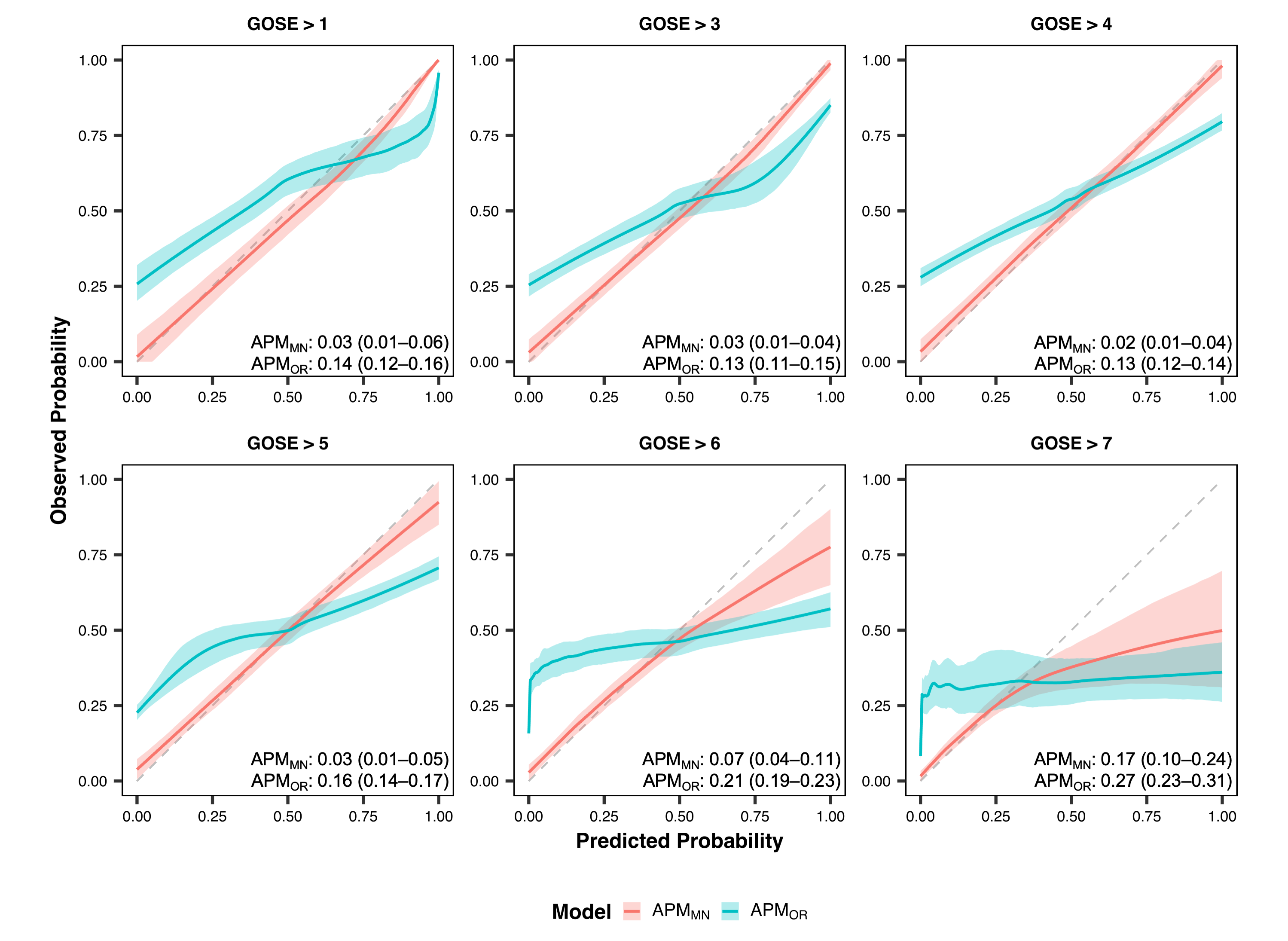

Supplement: S4 Fig — GOSE = Glasgow Outcome Scale–Extended at 6 months post-injury. Shaded areas are 95% confidence intervals derived using bias-corrected bootstrapping (1,000 resamples) to represent the variation across repeated k-fold cross-validation folds (20 repeats of 5 folds). The values in each panel correspond to the mean integrated calibration index (ICI) (95% confidence interval) at the given threshold. The diagonal dashed line represents the line of perfect calibration (ICI = 0). The APM types (APMMN and APMOR) are decoded in the Materials and methods and described in S2 Appendix. (TIF) [file pone.0270973.s008.tif]

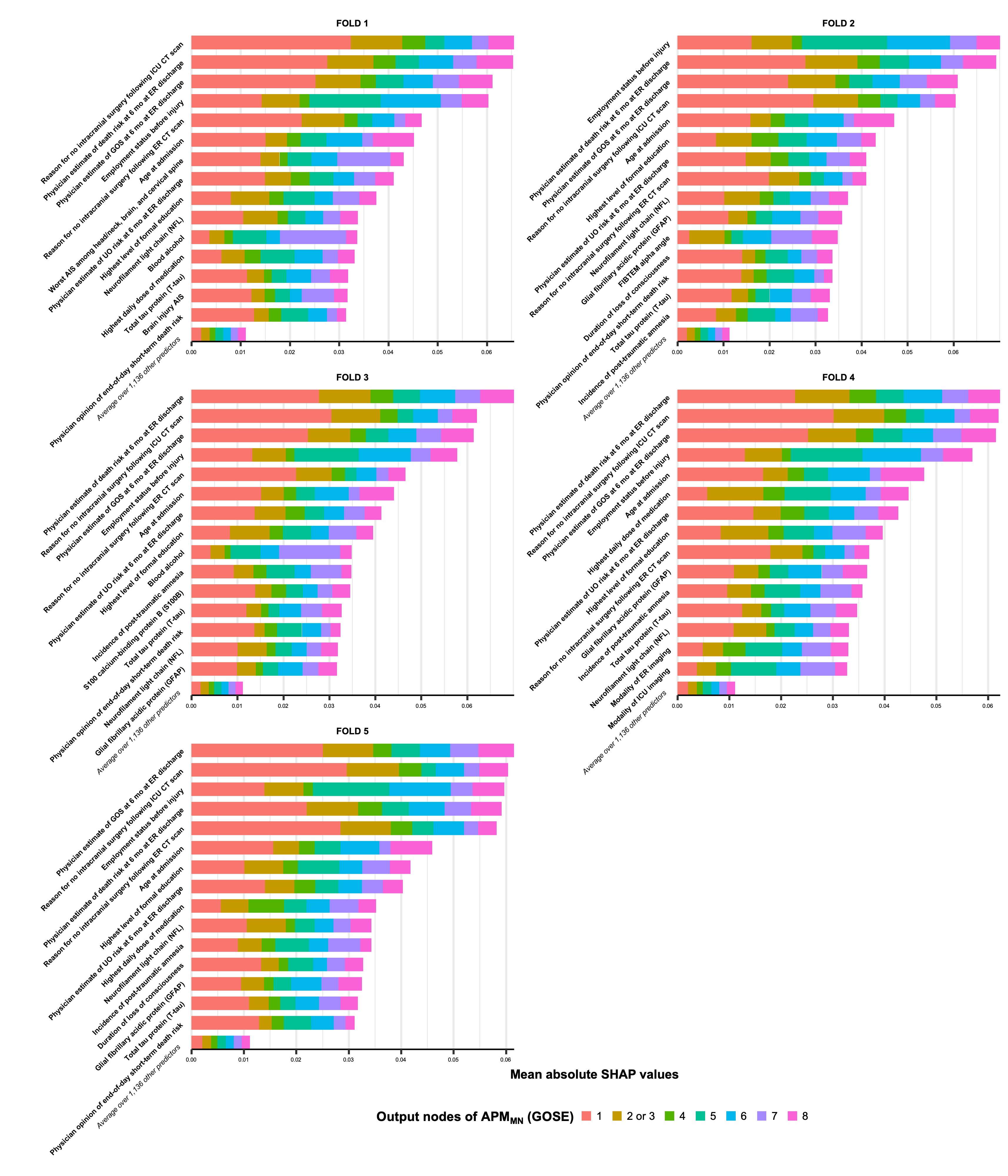

Supplement: S5 Fig — ICU = intensive care unit. CT = computerised tomography. ER = emergency room. GOS = Glasgow Outcome Scale (not extended). AIS = Abbreviated Injury Scale. UO = unfavourable outcome, defined by functional dependence (i.e., GOSE ≤ 4). FIBTEM = fibrin-based extrinsically activated test with tissue factor and cytochalasin D. GOSE = Glasgow Outcome Scale–Extended at 6 months post-injury. The mean absolute SHAP value is interpreted as the average magnitude of the relative additive contribution of a predictor’s most important token towards the predicted probability at each GOSE score for a single patient. (TIF) [file pone.0270973.s009.tif]

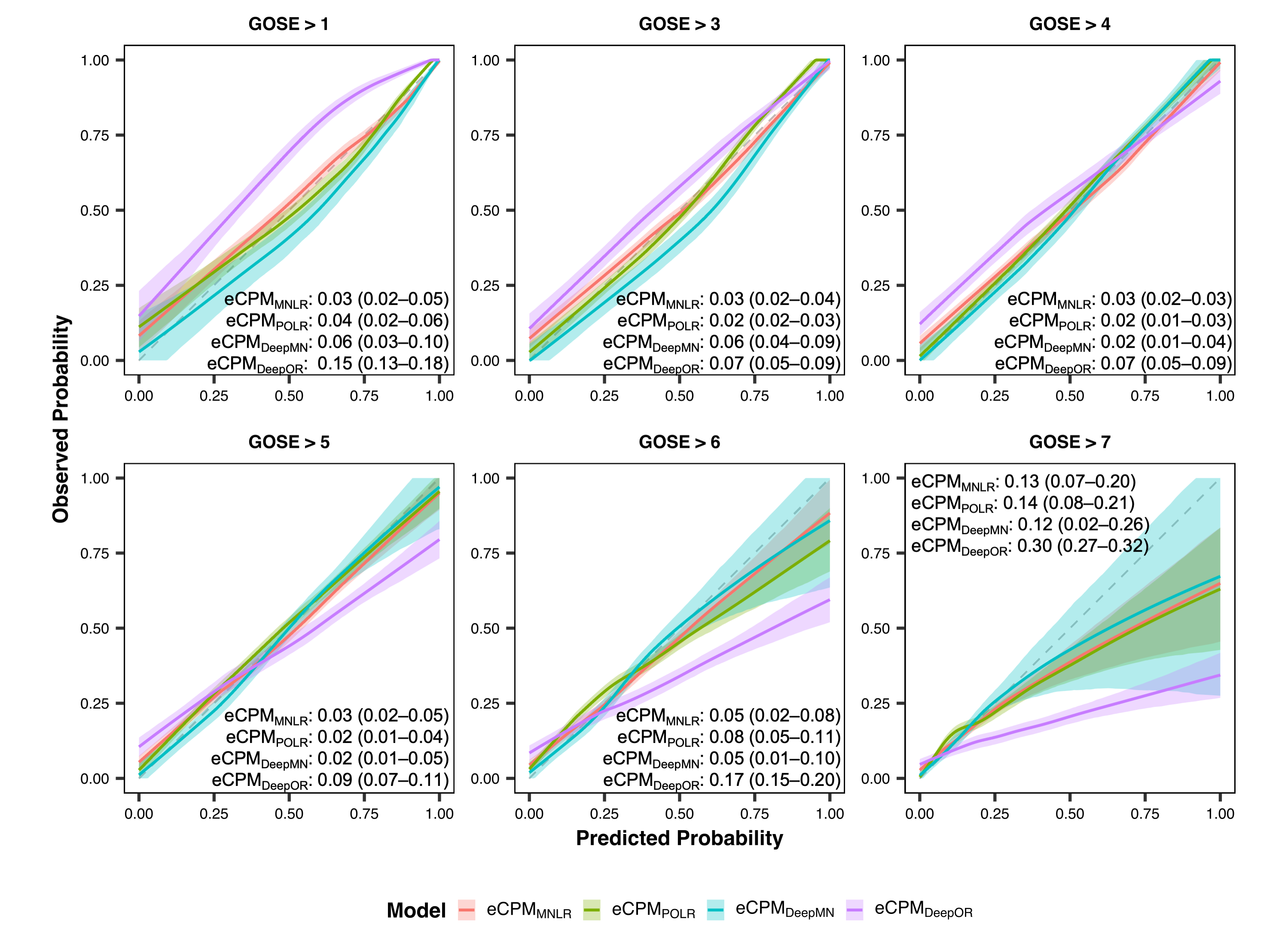

Supplement: S6 Fig — GOSE = Glasgow Outcome Scale–Extended at 6 months post-injury. Shaded areas are 95% confidence intervals derived using bias-corrected bootstrapping (1,000 resamples) to represent the variation across repeated k-fold cross-validation folds (20 repeats of 5 folds) and 100 missing value imputations. The values in each panel correspond to the mean integrated calibration index (ICI) (95% confidence interval) at the given threshold. The diagonal dashed line represents the line of perfect calibration (ICI = 0). The eCPM types (eCPMMNLR, eCPMPOLR, eCPMDeepMN, and eCPMDeepOR) are decoded in the Materials and methods and described in S1 Appendix. (TIF) [file pone.0270973.s010.tif]
