# Supplementary material for: The leap to ordinal: Detailed functional prognosis after traumatic brain injury with a flexible modelling approach
Source: PLoS One. 2022 Jul 5;17(7):e0270973. doi: 10.1371/journal.pone.0270973 (PMC9255749; doi:10.1371/journal.pone.0270973)
Supplement: S1 Table — (PDF) [file pone.0270973.s011.pdf]

S1 Table. Extended concise baseline predictors of the study population stratified by ordinal 6-month outcomes

Extended concise predictors	Overall	Glasgow Outcome Scale–Extended (GOSE) at 6 months post-injury							*p*-value[b]
	(*n* = 1550)	**1**	**2 or 3**	**4**	**5**	**6**	**7**	**8**	
		(*n* = 318)	(*n* = 262)	(*n* = 120)	(*n* = 227)	(*n* = 200)	(*n* = 206)	(*n* = 217)	
Age [years]	51 (31–66)	66 (50–76)	55 (36–68)	48 (29–61)	44 (31–56)	41 (27–53)	48 (31–65)	41 (24–61)	<0.0001
GCSm (*n*[a] = 1509)	5 (1–6)	2 (1–5)	3 (1–5)	5 (1–6)	5 (1–6)	5 (2–6)	5 (3–6)	6 (5–6)	<0.0001
(1) No response	484 (32.1%)	152 (50.0%)	104 (40.6%)	35 (29.9%)	63 (28.5%)	46 (23.6%)	47 (23.0%)	37 (17.5%)	
(2) Abnormal extension	54 (3.6%)	17 (5.6%)	20 (7.8%)	4 (3.4%)	6 (2.7%)	3 (1.5%)	2 (1.0%)	2 (0.9%)	
(3) Abnormal flexion	63 (4.2%)	14 (4.6%)	12 (4.7%)	8 (6.8%)	11 (5.0%)	8 (4.1%)	4 (2.0%)	6 (2.8%)	
(4) Withdrawal from stimulus	114 (7.6%)	27 (8.9%)	23 (9.0%)	8 (6.8%)	20 (9.0%)	21 (10.8%)	8 (3.9%)	7 (3.3%)	
(5) Movement localised to stimulus	305 (20.2%)	52 (17.1%)	47 (18.4%)	24 (20.5%)	50 (22.6%)	46 (23.6%)	44 (21.6%)	42 (19.8%)	
(6) Obeys commands	489 (32.4%)	42 (13.8%)	50 (19.5%)	38 (32.5%)	71 (32.1%)	71 (36.4%)	99 (48.5%)	118 (55.7%)	
Unreactive pupils (*n*[a] = 1465)									<0.0001
One	111 (7.6%)	31 (10.5%)	31 (12.3%)	7 (6.3%)	20 (9.3%)	5 (2.6%)	8 (4.1%)	9 (4.4%)	
Two	168 (11.5%)	84 (28.5%)	33 (13.0%)	8 (7.2%)	14 (6.5%)	8 (4.2%)	16 (8.2%)	5 (2.4%)	
Hypoxia	207 (13.4%)	60 (18.9%)	33 (12.6%)	14 (11.7%)	35 (15.4%)	33 (16.5%)	16 (7.8%)	16 (7.4%)	0.6272
Hypotension	210 (13.5%)	56 (17.6%)	51 (19.5%)	21 (17.5%)	32 (14.1%)	22 (11.0%)	15 (7.3%)	13 (6.0%)	0.0038
Marshall CT (*n*[a] = 1255)	VI (II–VI)	III (II–VI)	II (II–VI)	II (II–VI)	II (II–II)	II (II–III)	II (II–II)	VI (II–VI)	0.0386
No visible pathology (I)	118 (9.4%)	8 (3.3%)	11 (5.3%)	5 (5.2%)	17 (8.7%)	25 (15.2%)	24 (13.6%)	28 (16.5%)	
Diffuse injury II	592 (47.2%)	56 (22.8%)	84 (40.6%)	54 (56.2%)	92 (47.2%)	100 (60.6%)	103 (58.5%)	103 (60.6%)	
Diffuse injury III	108 (8.6%)	42 (17.1%)	17 (8.2%)	10 (10.4%)	14 (7.2%)	9 (5.5%)	6 (3.4%)	10 (5.9%)	
Diffuse injury IV	16 (1.3%)	7 (2.8%)	1 (0.5%)	1 (1.0%)	4 (2.1%)	1 (0.6%)	1 (0.6%)	1 (0.6%)	
Mass lesion (V & VI)	421 (33.5%)	133 (54.0%)	94 (45.4%)	26 (27.1%)	68 (34.9%)	30 (18.2%)	42 (23.9%)	28 (16.5%)	
tSAH (*n*[a] = 1254)	957 (76.3%)	221 (90.2%)	176 (84.2%)	73 (76.0%)	150 (76.9%)	106 (63.9%)	125 (71.4%)	106 (63.1%)	0.4429
EDH (*n*[a] = 1257)	244 (19.4%)	31 (12.7%)	32 (15.3%)	21 (21.9%)	46 (23.6%)	32 (19.3%)	42 (23.9%)	40 (23.5%)	0.0035
Glucose [mmol/L] (*n*[a] = 1062)	7.7 (6.6–9.4)	8.8 (7.3–11)	8.0 (6.5–9.8)	7.6 (6.5–9.3)	7.8 (6.6–9.6)	7.7 (6.5–8.7)	7.3 (6.3–8.5)	7.1 (6.3–8.1)	0.0123
Hb [g/dL] (*n*[a] = 1140)	13 (12–14)	13 (11–14)	13 (11–14)	14 (12–14)	13 (12–14)	14 (12–15)	13 (12–15)	14 (13–15)	0.3044
Retired (*n*[a] = 1312)	353 (26.9%)	136 (61.3%)	74 (33.6%)	23 (22.1%)	12 (5.9%)	13 (7.3%)	52 (28.1%)	43 (21.8%)	0.0644
Highest formal education (*n*[a] = 1110)									0.4897
None	15 (1.4%)	3 (2.4%)	4 (2.1%)	2 (2.0%)	2 (1.1%)	2 (1.2%)	0 (0%)	2 (1.1%)	
In degree program	26 (2.3%)	0 (0%)	5 (2.6%)	0 (0%)	4 (2.1%)	7 (4.1%)	4 (2.5%)	6 (3.4%)	
Primary school	155 (14.0%)	31 (24.6%)	44 (23.3%)	14 (13.9%)	17 (8.9%)	16 (9.5%)	14 (8.8%)	19 (10.9%)	

Secondary school	458 (41.3%)	50 (39.7%)	63 (33.3%)	46 (45.5%)	80 (42.1%)	59 (34.9%)	75 (46.9%)	85 (48.6%)	
Technical certificate	235 (21.2%)	16 (12.7%)	38 (20.1%)	21 (20.8%)	57 (30.0%)	43 (25.4%)	32 (20.0%)	28 (16.0%)	
University degree	221 (19.9%)	26 (20.6%)	35 (18.5%)	18 (17.8%)	30 (15.8%)	42 (24.9%)	35 (21.9%)	35 (20.0%)	
GFAP [ng/mL] (n^a = 1247)	17 (6–46)	48 (15–96)	32 (11–61)	17 (6–43)	13 (5–30)	13 (5–30)	10 (3–23)	9 (3–22)	0.0005
T-tau [pg/mL] (n^a = 1248)	8 (4–19)	17 (7–38)	12 (6–23)	9 (5–19)	7 (3–14)	7 (3–13)	5 (3–12)	6 (3–11)	0.2568
S100B [ng/mL] (n^a = 1267)	0.3 (.2–.6)	0.6 (.3–1.3)	0.4 (.2–.6)	0.3 (.2–.6)	0.3 (.2–.4)	0.2 (.2–.4)	0.2 (.1–.5)	0.2 (.1–.3)	0.1929
NFL [pg/mL] (n^a = 1247)	55 (28–127)	121 (51–268)	85 (46–150)	61 (32–150)	48 (28–87)	41 (21–87)	30 (17–60)	35 (19–74)	0.3054
PTA (n^a = 1530)	187 (12.2%)	5 (1.6%)	15 (5.8%)	10 (8.5%)	43 (19.3%)	33 (16.8%)	50 (24.4%)	31 (14.4%)	0.0010
Worst head/neck, brain, or cervical spine AIS (n^a = 1523)									0.0001
(1) Minor	50 (3.2%)	6 (1.9%)	3 (1.1%)	5 (4.2%)	5 (2.2%)	4 (2.0%)	16 (7.8%)	11 (5.1%)	
(2) Moderate	31 (2.0%)	3 (0.9%)	3 (1.1%)	0 (0%)	5 (2.2%)	4 (2.0%)	8 (3.9%)	8 (3.7%)	
(3) Serious	112 (7.2%)	6 (1.9%)	6 (2.3%)	7 (5.8%)	21 (9.3%)	19 (9.5%)	25 (12.1%)	28 (12.9%)	
(4) Severe	484 (31.2%)	63 (19.8%)	54 (20.6%)	37 (30.8%)	71 (31.3%)	78 (39.0%)	87 (42.2%)	94 (43.3%)	
(5) Critical	846 (54.6%)	216 (67.9%)	195 (74.4%)	70 (58.3%)	125 (55.1%)	94 (47.0%)	70 (34.0%)	76 (35.0%)	
(6) Not survivable	27 (1.7%)	24 (7.5%)	1 (0.4%)	1 (0.8%)	0 (0%)	1 (0.5%)	0 (0%)	0 (0%)	

Data are median (IQR) for continuous characteristics and *n* (% of column group) for categorical characteristics. Units of characteristics are provided in square brackets. GCSm=motor component score of the Glasgow Coma Scale. Marshall CT=Marshall computerised tomography classification. tSAH=traumatic subarachnoid haemorrhage. EDH=extradural haematoma. Glu=glucose. Hb=haemoglobin. GFAP=glial fibrillary acidic protein. T-tau=total tau protein. S100B=S100 calcium-binding protein B. NFL=neurofilament light chain. PTA=incidence of post-traumatic amnesia. AIS=abbreviated injury scale.

[a]Limited sample size of non-missing values for characteristic.

[b]*p*-values are determined from proportional odds logistic regression analysis trained on all concise predictors concurrently [19] and are combined across 100 missing value imputations via *z*-transformation [29]. For categorical variables with $k > 2$ categories (e.g., GCSm), *p*-values were calculated with a likelihood ratio test (with k-1 degrees of freedom) on POLR.
