# Supplementary material for: The leap to ordinal: Detailed functional prognosis after traumatic brain injury with a flexible modelling approach
Source: PLoS One. 2022 Jul 5;17(7):e0270973. doi: 10.1371/journal.pone.0270973 (PMC9255749; doi:10.1371/journal.pone.0270973)
Supplement: S2 Table — (PDF) [file pone.0270973.s012.pdf]

S2 Table. Ordinal concise-predictor-based model (CPM) discrimination and calibration performance

Metric	Threshold	Model			
		CPM_{MNLR}	CPM_{POLR}	CPM_{DeepMN}	CPM_{DeepOR}
Ordinal *c*-index (ORC)		0.69 (0.67–0.70)	0.69 (0.68–0.70)	0.70 (0.68–0.71)	0.59 (0.58–0.61)
Somers' D_{xy}		0.43 (0.41–0.45)	0.43 (0.41–0.46)	0.44 (0.41–0.48)	0.23 (0.20–0.26)
Threshold-level dichotomous *c*-index[a]		0.77 (0.75–0.78)	0.77 (0.75–0.78)	0.76 (0.74–0.78)	0.76 (0.73–0.78)
	GOSE > 1	0.83 (0.81–0.85)	0.83 (0.81–0.84)	0.83 (0.80–0.86)	0.82 (0.79–0.85)
	GOSE > 3	0.81 (0.79–0.83)	0.81 (0.79–0.82)	0.80 (0.78–0.83)	0.80 (0.77–0.82)
	GOSE > 4	0.78 (0.76–0.80)	0.78 (0.76–0.79)	0.77 (0.74–0.80)	0.77 (0.74–0.79)
	GOSE > 5	0.76 (0.74–0.77)	0.76 (0.74–0.77)	0.75 (0.72–0.78)	0.74 (0.71–0.77)
	GOSE > 6	0.72 (0.70–0.74)	0.71 (0.69–0.73)	0.71 (0.68–0.74)	0.71 (0.67–0.74)
	GOSE > 7	0.72 (0.69–0.74)	0.73 (0.70–0.75)	0.71 (0.67–0.75)	0.71 (0.67–0.75)
Threshold-level calibration slope[a]		0.85 (0.78–0.91)	0.94 (0.88–1.01)	0.98 (0.81–1.12)	0.90 (0.79–1.02)
	GOSE > 1	0.92 (0.84–1.00)	1.13 (1.04–1.23)	0.95 (0.78–1.10)	1.01 (0.85–1.18)
	GOSE > 3	0.92 (0.85–1.00)	1.14 (1.05–1.23)	0.97 (0.80–1.12)	0.95 (0.83–1.09)
	GOSE > 4	0.91 (0.84–1.00)	0.99 (0.91–1.08)	1.06 (0.86–1.23)	0.93 (0.80–1.06)
	GOSE > 5	0.88 (0.80–0.97)	0.90 (0.82–0.99)	1.01 (0.78–1.21)	0.90 (0.76–1.06)
	GOSE > 6	0.81 (0.71–0.91)	0.71 (0.63–0.80)	0.98 (0.73–1.20)	0.86 (0.67–1.06)
	GOSE > 7	0.64 (0.50–0.80)	0.77 (0.67–0.88)	0.92 (0.69–1.18)	0.78 (0.57–1.02)

Data represent mean (95% confidence interval) for the CPM based on a given metric. Interpretations for each metric are provided in **Materials and methods**. Mean and confidence interval values were derived using bias-corrected bootstrapping (1,000 resamples) and represent the variation across repeated *k*-fold cross-validation folds (20 repeats of 5 folds) and 100 missing value imputations. GOSE=Glasgow Outcome Scale – Extended at 6 months post-injury. The CPM types (CPM_{MNLR}, CPM_{POLR}, CPM_{DeepMN}, and CPM_{DeepOR}) are decoded in the **Materials and methods** and described in **S1 Appendix**.
[a]Values in these rows correspond to the unweighted average across all GOSE thresholds.
