# Supplementary material for: The leap to ordinal: Detailed functional prognosis after traumatic brain injury with a flexible modelling approach
Source: PLoS One. 2022 Jul 5;17(7):e0270973. doi: 10.1371/journal.pone.0270973 (PMC9255749; doi:10.1371/journal.pone.0270973)
Supplement: S3 Table — (PDF) [file pone.0270973.s013.pdf]

S3 Table. Ordinal all-predictor-based model (APM) discrimination and calibration performance

Metric	Threshold	Model	
		APM_{MN}	APM_{OR}
Ordinal *c*-index (ORC)		0.76 (0.74–0.77)	0.66 (0.65–0.68)
Somers' D_{xy}		0.57 (0.54–0.60)	0.37 (0.33–0.40)
Threshold-level dichotomous *c*-index[a]		0.82 (0.80–0.83)	0.78 (0.76–0.80)
	GOSE > 1	0.90 (0.88–0.92)	0.83 (0.81–0.85)
	GOSE > 3	0.86 (0.84–0.88)	0.82 (0.80–0.84)
	GOSE > 4	0.83 (0.80–0.85)	0.80 (0.78–0.82)
	GOSE > 5	0.80 (0.78–0.83)	0.78 (0.75–0.80)
	GOSE > 6	0.76 (0.73–0.79)	0.74 (0.71–0.77)
	GOSE > 7	0.75 (0.72–0.79)	0.71 (0.68–0.75)
Threshold-level calibration slope[a]		0.84 (0.76–0.91)	0.13 (0.12–0.15)
	GOSE > 1	0.98 (0.86–1.10)	0.35 (0.31–0.38)
	GOSE > 3	0.90 (0.80–1.02)	0.18 (0.16–0.21)
	GOSE > 4	0.89 (0.79–1.00)	0.10 (0.09–0.12)
	GOSE > 5	0.82 (0.72–0.94)	0.07 (0.06–0.09)
	GOSE > 6	0.74 (0.62–0.87)	0.06 (0.05–0.07)
	GOSE > 7	0.68 (0.54–0.83)	0.05 (0.04–0.06)

Data represent mean (95% confidence interval) for the APM based on a given metric. Interpretations for each metric are provided in **Materials and methods**. Mean and confidence interval values were derived using bias-corrected bootstrapping (1,000 resamples) and represent the variation across repeated *k*-fold cross-validation folds (20 repeats of 5 folds). GOSE=Glasgow Outcome Scale – Extended at 6 months post-injury. The APM types (APM_{MN} and APM_{OR}) are decoded in the **Materials and methods** and described in **S2 Appendix**.

[a]Values in these rows correspond to the unweighted average across all GOSE thresholds.
