# Supplementary material for: The leap to ordinal: Detailed functional prognosis after traumatic brain injury with a flexible modelling approach
Source: PLoS One. 2022 Jul 5;17(7):e0270973. doi: 10.1371/journal.pone.0270973 (PMC9255749; doi:10.1371/journal.pone.0270973)
Supplement: S4 Table — (PDF) [file pone.0270973.s014.pdf]

S4 Table. Ordinal extended concise-predictor-based model (eCPM) discrimination and calibration performance

Metric	Threshold	Model			
		$eCPM_{MNLR}$	$eCPM_{POLR}$	$eCPM_{DeepMN}$	$eCPM_{DeepOR}$
Ordinal *c*-index (ORC)		0.72 (0.71–0.73)	0.71 (0.70–0.72)	0.73 (0.71–0.74)	0.67 (0.65–0.68)
Somers' D_{xy}		0.50 (0.48–0.52)	0.47 (0.45–0.49)	0.50 (0.46–0.54)	0.38 (0.35–0.41)
Threshold-level dichotomous *c*-index[a]		0.79 (0.78–0.80)	0.79 (0.78–0.80)	0.79 (0.77–0.81)	0.77 (0.76–0.79)
	GOSE > 1	0.86 (0.84–0.87)	0.85 (0.84–0.87)	0.86 (0.83–0.88)	0.85 (0.82–0.87)
	GOSE > 3	0.84 (0.83–0.86)	0.84 (0.83–0.85)	0.84 (0.82–0.86)	0.83 (0.81–0.85)
	GOSE > 4	0.82 (0.80–0.83)	0.81 (0.80–0.83)	0.81 (0.79–0.83)	0.80 (0.77–0.82)
	GOSE > 5	0.77 (0.75–0.79)	0.77 (0.76–0.79)	0.77 (0.74–0.80)	0.76 (0.73–0.78)
	GOSE > 6	0.75 (0.73–0.77)	0.73 (0.71–0.75)	0.74 (0.70–0.77)	0.72 (0.69–0.75)
	GOSE > 7	0.72 (0.70–0.75)	0.73 (0.70–0.75)	0.72 (0.68–0.76)	0.70 (0.66–0.74)
Threshold-level calibration slope[a]		0.75 (0.70–0.81)	0.89 (0.83–0.95)	1.00 (0.78–1.14)	0.59 (0.51–0.67)
	GOSE > 1	0.81 (0.75–0.89)	0.97 (0.87–1.10)	0.98 (0.78–1.14)	1.04 (0.90–1.20)
	GOSE > 3	0.83 (0.77–0.90)	1.12 (1.04–1.23)	1.05 (0.81–1.20)	0.79 (0.68–0.90)
	GOSE > 4	0.81 (0.75–0.89)	1.02 (0.94–1.11)	1.10 (0.85–1.27)	0.60 (0.52–0.69)
	GOSE > 5	0.75 (0.67–0.82)	0.86 (0.78–0.94)	1.01 (0.76–1.22)	0.47 (0.38–0.56)
	GOSE > 6	0.72 (0.63–0.81)	0.69 (0.62–0.77)	0.97 (0.70–1.20)	0.36 (0.27–0.46)
	GOSE > 7	0.58 (0.48–0.69)	0.68 (0.59–0.77)	0.89 (0.61–1.18)	0.28 (0.16–0.40)

Data represent mean (95% confidence interval) for the eCPM based on a given metric. Interpretations for each metric are provided in **Materials and methods**. Mean and confidence interval values were derived using bias-corrected bootstrapping (1,000 resamples) and represent the variation across repeated *k*-fold cross-validation folds (20 repeats of 5 folds) and 100 missing value imputations. GOSE=Glasgow Outcome Scale – Extended at 6 months post-injury. The eCPM types ($eCPM_{MNLR}$, $eCPM_{POLR}$, $eCPM_{DeepMN}$, and $eCPM_{DeepOR}$) are decoded in the **Materials and methods** and described in **S1 Appendix**.
[a]Values in these rows correspond to the unweighted average across all GOSE thresholds.
